# Dual Stimuli-Responsive Copper Nanoparticles Decorated SBA-15: A Highly Efficient Catalyst for the Oxidation of Alcohols in Water

**DOI:** 10.3390/nano10102051

**Published:** 2020-10-16

**Authors:** Anju Maria Thomas, Jerome Peter, Saravanan Nagappan, Anandhu Mohan, Chang-Sik Ha

**Affiliations:** Department of Polymer Science and Engineering, Pusan National University, Busan 46241, Korea; anju.05u0@gmail.com (A.M.T.); jestro.12@gmail.com (J.P.); saravananagappa@gmail.com (S.N.); anandhumohangcm@gmail.com (A.M.)

**Keywords:** mesoporous silica, stimuli-responsive polymer, copper nanoparticles, oxidation of alcohols

## Abstract

In the present work, a temperature and pH-responsive hybrid catalytic system using copolymer-capped mesoporous silica particles with metal nanoparticles is proposed. The poly(2-(dimethylamino)ethyl methacrylate)(DMAEMA)-*co*-N-*tert*-butyl acrylamide) (TBA)) shell on mesoporous silica SBA-15 was obtained through free radical polymerization. Then, copper nanoparticles (CuNPs) decorated SBA-15/copolymer hybrid materials were synthesized using the NaBH_4_ reduction method. SBA-15 was functionalized with trimethoxylsilylpropyl methacrylate (TMSPM) and named TSBA. It was found that the CuNPs were uniformly dispersed in the mesoporous channels of SBA-15, and the hybrid catalyst exhibited excellent catalytic performance for the selective oxidation of different substituted benzyl alcohols in water using H_2_O_2_ as an oxidant at room temperature. The dual (temperature and pH-) responsive behaviors of the CuNPs/p(DMAEMA-*co*-TBA)/TSBA catalyst were investigated using the dynamic light scattering technique. The conversion of catalytic products and selectivity were calculated using gas chromatographic techniques, whereas the molecular structure of the products was identified using ^1^H and ^13^C nuclear magnetic resonance (NMR) spectroscopy. The catalyst showed excellent catalytic activity toward the oxidation of alcohol to aldehyde in an aqueous medium below the lower critical solution temperature (LCST) and pKa values (7–7.5) of the copolymer. The main advantages of the hybrid catalyst, as compared to the existing catalysts, are outstanding alcohol conversion (up to 99%) for a short reaction time (1 h), small amount of the catalyst (5 mg), and good recyclability equal to at least five times.

## 1. Introduction

Selective oxidation of benzyl alcohol to benzaldehyde is essential in perfumery and food industries. The eco-friendly oxidation of benzyl alcohol can be processed using hydrogen peroxide as an oxidant [1,2,3]. A variety of metal nanoparticle-supported materials have been used as catalysts for the oxidation of benzyl alcohol including supported catalysts such as Au/CeO_2_ [4], Au/CuO [5], Pd/hydrotalcite [6], and Pd/hydroxyapatite [7] nanoparticles. However, precious metals used for the catalysts are usually not so cheap. In this sense, copper-supported catalysts may be an alternative [8,9,10,11,12]. Few works on the use of copper or copper oxide nanoparticles-based catalysts were, however, reported in benzyl alcohol oxidation [8,9,10,11,12,13]. Moreover, the use of copper nanoparticles (CuNPs) is restricted by Cu’s inherent instability under atmospheric conditions, which makes it prone to oxidation. Some supporting materials increase the stability of CuNPs by altering their sensitivity to oxygen, water, and other chemical entities, which has encouraged the exploration of alternative Cu-based NPs with more complex structures [12,14]. 

Mesoporous silica material-supported catalysts have been proven to be the most ideal catalysts because of their high surface area, wall thickness, high porosity, and large and uniform channel size. To prevent the agglomeration of CuNPs, the use of a stabilizing agent is essential for the synthesis of CuNPs [15,16,17]. Although the wet impregnation method is an effective method for incorporating metals on a silica surface, that method usually results in non-uniform dispersion. This becomes particularly troublesome because in those mesoporous supports, pores can be completely blocked, reducing the active surface areas. To ensure better incorporation of metal nanoparticles, functional groups are often decorated onto the mesoporous silica surface through a surface modification process [17,18,19]. The grafted functional groups such as amine, thiol, cyano, vinyl, alkyl, and phenyl can act as anchors to enhance the interaction between the silica wall and the metal precursor [20,21,22,23]. Here, we used trimethoxy silyl propyl methacrylate (TMSPM) for the surface functionalization of SBA-15. Then, we have covered the surface of SBA-15 with poly(N,N-dimethylaminoethyl methacrylate) (p(DMAEMA)), which is an example of a thermo-responsive polymer and it also possesses pH sensitivity. The lower critical solution temperature (LCST) of p(DMAEMA) is dependent on the pH. At high pH, the amino groups are protonated and the electrostatic repulsions between the polymeric chains impede the collapse [24,25,26]. One of the interesting research areas for stimuli-responsive materials is to tune the phase transition temperatures near the ambient region (0–100 °C). The LCST will be lowered when the polymer is copolymerized with a hydrophobic co-monomer, while the LCST will be increased by adding a hydrophilic co-monomer. Herein, we used *ter*t-butyl acrylamide (TBA) as a co-monomer with PDMAEMA to lower the LCST value. The LCST of DMAEMA is in the range of 45–50 °C. After copolymerization with TBA, LCST reaches approximately 20–25 °C. The thermo-responsive behavior of the catalyst can be explained by the polymer covering the surface of mesoporous silicas, which act as a gate to the pore entrance [27,28]. Below the LCST, the polymer is swollen, so the gate will open, and the reactant molecules can easily penetrate the pores and react with the metal nanoparticles inside the pores. Above the LCST, the polymer layer on the SBA-15 surface was shrunken, so the gate was closed, and it was difficult for the reactants to approach the metal side. Polymer-grafted mesoporous silicas can be used in the field of drug delivery, catalysis, and separation, particularly for the oxidation of various alcohols. In this study, we synthesized an organic–inorganic nanohybrid catalyst based on copper nanoparticles-decorated copolymer-grafted mesoporous SBA-15 silica material. This study aims to investigate the effect of the dual stimuli (temperature and pH)-responsive behavior of the catalyst used for the oxidation of benzyl alcohol in a water medium using H_2_O_2_ as an oxidant. We also tested the reusability of the catalyst.

## 2. Materials and Methods 

### 2.1. Synthesis of p(DMAEMA-co-TBA)/TSBA Hybrid Particles

The copolymerization reaction was performed in a three-neck flask equipped with a reflux condenser to synthesize p(DMAEMA-*co*-TBA). During the experiment, the reaction was homogenized using a magnetic stirrer. A solution of 0.6 g of DMAEMA and 0.4 g of TBA was placed in a round bottom flask. Ethanol and water mixtures were used as solvents under nitrogen flow. Then, 0.2 g of modified SBA-15 (named TSBA, which is SBA-15 functionalized with trimethoxyl silyl propyl methacrylate (TMSPM); the detailed experimental procedure is provided in the Supplementary Data) was added to the reaction mixture. After 1 h, 0.027 g of potassium persulfate (K_2_S_2_O_8_) initiator was added. The mixture was heated to 80 °C, and the polymerization continued for 7 h, followed by centrifugation at 10,000 rpm and washed thoroughly with water and ethanol. The mixture was then dried at 50 °C under a vacuum to obtain the product denoted as p(DMAEMA-*co*-TBA)/TSBA. 

### 2.2. Loading of Copper Nanoparticles (CuNPs) into p(DMAEMA-co-TBA)/TSBA

0.05 g of poly(DMAEMA*-co-*TBA*)/*TSBA was dispersed in water after sonication for 15 min. The suspension was continuously stirred for 2 h at room temperature to achieve a complete dispersion of SBA-15. Then, a 0.01 M solution of Cu(II)Br was added, and the reaction was stirred for 6 h under a nitrogen atmosphere. Finally, the metal precursor was reduced to metal nanoparticles through reduction with the addition of 0.1 M NaBH_4_ solution under mild stirring. The original clear white solution turned black after the reaction. The reaction was stirred for 1 h. Then, the catalyst was retrieved through filtration, washed carefully with water, and dried at 50 °C. The obtained product was named CuNPs/p(DMAEMA-*co-*TBA)/TSBA. Chemicals for the synthesis of materials in this Section 2 and their basic characterization methods are described in the Supplementary Materials.

### 2.3. Catalysis—Oxidation of Alcohols

The synthesized CuNPs-supported hybrid catalyst was tested for the oxidation of alcohols using benzyl alcohol as a model substrate and optimized using various parameters such as the oxidant, solvent, and temperature. In a typical experiment, 5 mg of the catalyst was dispersed in 5 mL of deionized water and stirred for 10 min. Then, benzyl alcohol (1 mmol) was added, and nitrogen purging was conducted for 2 min, followed by the dropwise addition of hydrogen peroxide (1 mmol). After the addition of H_2_O_2_ the pH of the system was found to be 6. The reaction mixture was stirred at an ambient temperature. After the reaction was completed, the mixture was filtered, separated, and extracted using diethyl ether (2 × 10 mL). The catalytic products were analyzed using gas chromatography (GC). Similar reaction conditions and methodology were used for other alcoholic substrates, and the conversion and selectivity were estimated. The resulting products were further purified using column chromatography, and their molecular structures were identified by ^1^H and ^13^C nuclear magnetic resonance (NMR) spectra using CDCl_3_ solvent.

## 3. Results and Discussion

### 3.1. Characterization of Materials

**Figure 1:**
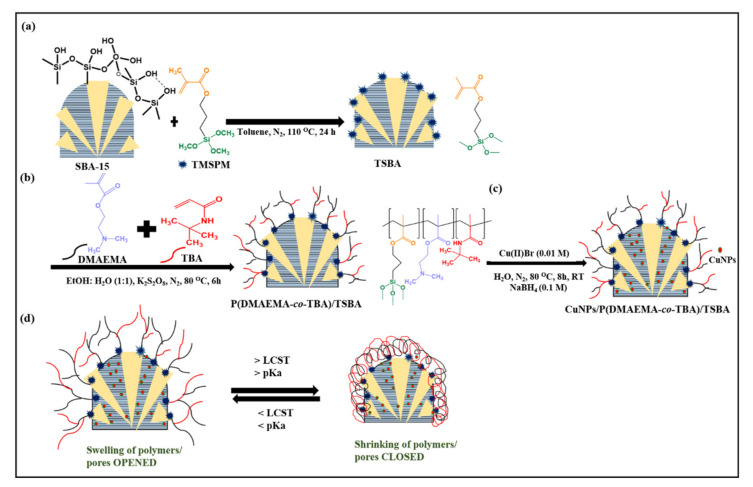
(**a**) Modification of surface hydroxyl groups on the SBA-15 with silane group of trimethoxy silyl propyl methacrylate (TMSPM); (**b**) structure of the copolymer grafted surface of SBA-15; (**c**) incorporation of copper nanoparticles (CuNPs) on to the p(DAMEAM-*co*-TBA)/TSBA (where, TSBA is the modified SBA-15); (**d**) closing of opening of mesopores with respect to the responsive behavior of the copolymer. Chemicals for the syntheses of materials in Section 2 and their basic characterization methods are described in the Supplementary Materials.

The synthesis routes for the grafting of the copolymer on the SBA-15 particles are shown in Figure 1. In the first step, the external surface of SBA-15 was functionalized with TMSPM (Figure 1a). In the second step, copolymerization of the grafted particles with DMAEMA and TBA was conducted (Figure 1b). The structure of the copolymer and its grafting procedure are shown in Figure 1b. Finally, the CuNPs were loaded into the p(DMAEMA-*co*-TBA)/TSBA (Figure 1c). The temperature- and pH-responsive behavior of the catalyst depends upon the shrinking/swelling and protonation/deprotonation environment of the dimethyl amino groups (pKa of 7–7.5) of the copolymer chain upon heating/cooling. At low pH levels, the amino groups in DMAEMA have a positive charge; therefore, maximum swelling occurs due to the repulsion of like charges generated in the polymer, and the pores are found to be in the relaxed open state. In contrast, the closing of the windows of mesopores occurs at high pH conditions due to the deprotonation of amino groups (Figure 1d).

**Figure 2:**
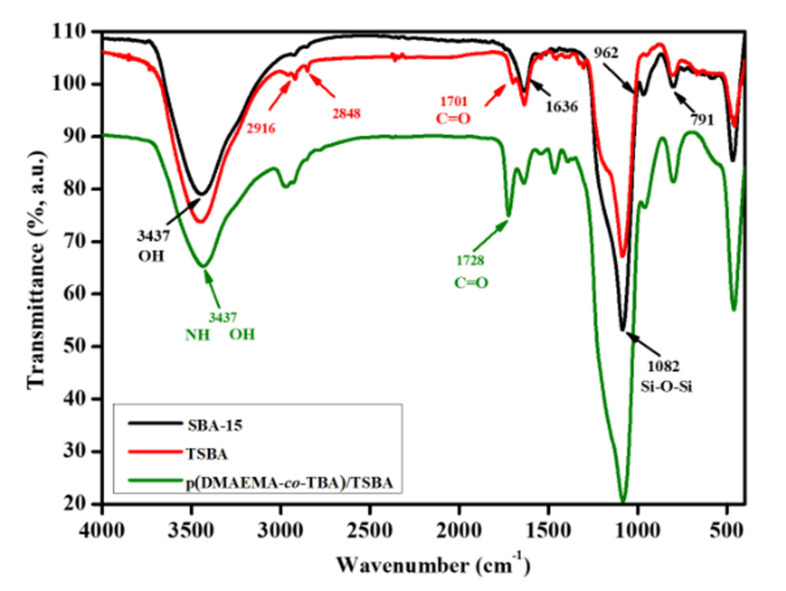
FT-IR spectra of SBA-15, TSBA, and p(DMAEMA-*co*-TBA)/TSBA.

Figure 2 illustrates the Fourier transform infrared (FT-IR) spectra of SBA-15, TSBA, and p(DMAEMA-*co*-TBA)/TSBA. The detailed band assignment of SBA-15 and functionalized mesoporous silica has been reported in previous literatures [26,27]. The broad bands of adsorption at approximately 3437 cm^−1^ can be assigned to the water molecules hydrogen-bonded to each other and to Si–OH groups. The bands at approximately 1636 cm^−1^ are due to the bending vibrations of O–H bonds in H_2_O groups, overlapping with stretching vibrations of C–O–C. The bands near 1082 cm^−1^ correspond to the Si–O–Si asymmetric stretching vibrations, overlapped with Si–O–C, C–O–C, and Si–C vibrations. The bands at 962 cm^−1^ are attributed to the stretching vibrations of free Si–OH groups on the amorphous solid sample surface, while CO stretching vibration bonds are also overlapped in the range. The characteristic peak at 1701 cm^−1^ for the modified SBA-15 could be assigned to C=O stretching, and the peaks at 2916 cm^−1^ and 2848 cm^−1^ correspond to the C–H stretching and bending vibration, respectively, present in TMSPM. The sharp band at 1728 cm^−1^ suggests the existence of the C=O group in DMAEMA. However, there is a broadening of the band intensity at 3437 cm^−1^ because of the presence of NH and OH functional groups, indicating the successful attachment of TBA and DMAEMA to the surface of SBA-15.

**Figure 3:**
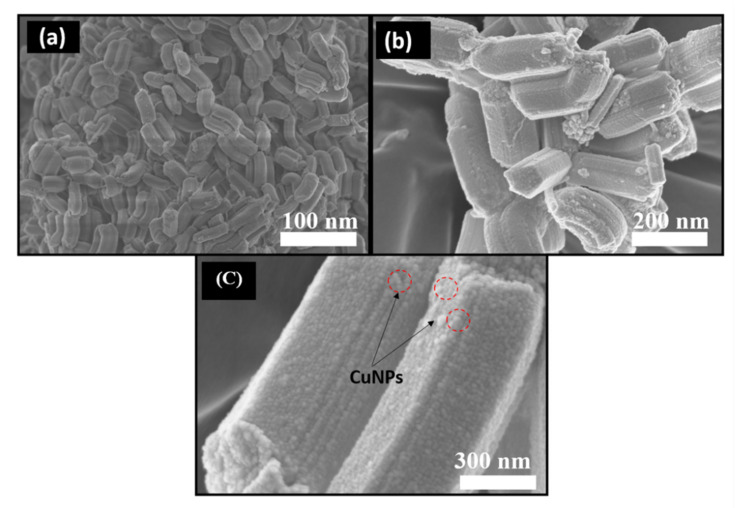
Field emission scanning electron microscopy (FESEM) images of (**a**) SBA-15; (**b**) p(DMAEMA-*co*-TBA)/TSBA; (**c**) CuNPs/p(DMAEMA-*co*-TBA)/TSBA.

Field emission scanning electron microscopy (FESEM) images of rod-shaped SBA-15, p(DMAEMA-*co*-TBA)/TSBA, and CuNPs/p(DMAEMA-*co*-TBA)/TSBA are shown in Figure 3. SBA-15 shows long rod-shaped particles with lengths and widths of 1 μm and 130 nm, respectively, as reported in previous literature [26] (Figure 3a). The surface of SBA-15 was quite smooth and clear, without any impurities. The interaction between the surfactant and the precursor is responsible for the formation of mesoporous materials during the self-assembly process. The exterior surface became rougher after polymer coating, which confirms the covering of SBA-15 with the copolymer. (Figure 3b). A small amount of CuNP loading does not affect the morphology of the mesoporous silica. The CuNPs/p(DMAEMA-*co*-TBA)/TSBA also displayed SBA-15 morphology (Figure 3c). The small particles observed on the surface confirmed the presence of agglomerated CuNPs.

**Figure 4:**
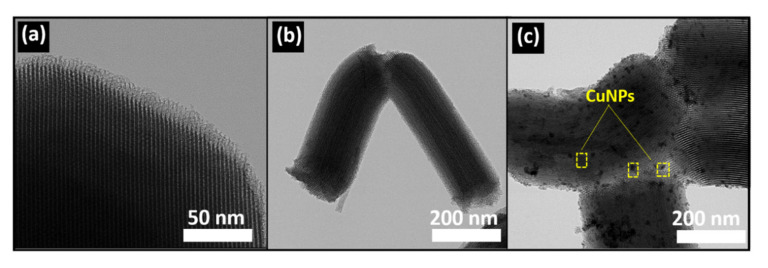
High-resolution transmission electron microscopy (HRTEM) images of (**a**) SBA-15; (**b**) p(DMAEMA-*co*-TBA)/TSBA, and (**c**) CuNPs/p(DMAEMA-*co*-TBA)/TSBA.

**Figure 5:**
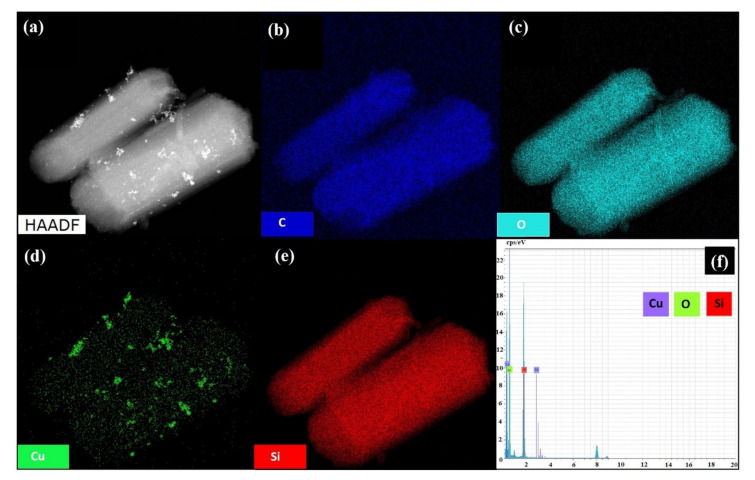
HAADF-TEM (**a**) Energy-dispersive X-ray spectroscopy (EDX) mapping images; (**b**–**e**) and spectrum; (**f**) of CuNPs/p(DMAEMA-*co*-TBA)/TSBA.

Figure 4 displays high-resolution transmission electron microscopy (HRTEM) images of SBA-15, p(DMAEMA-*co*-TBA)/TSBA, and CuNPs/p(DMAEMA-*co*-TBA)/TSB. The images clearly show the well-ordered arrangement of mesopores in SBA-15 for all three samples. The mesoporous structure was maintained even after modification, which confirms that the modification does not affect the pore structure of SBA-15. After polymerization with DMAEMA and TBA, the material preserved the mesoporosity (Figure 4b). These images further support the structural integrity of the silicate matrix, which was maintained even after metal incorporation. The presence of dark spots confirms the presence of CuNPs both inside and outside of the mesopores (Figure 4c). HRTEM images, together with energy dispersive X-ray (EDX) mapping, indicate the presence of CuNPs in the CuNPs/p(DMAEMA-*co*-TBA)/TSBA catalyst. The weight % of the loaded CuNPs was found to be 1.02% in the catalyst (Figure 5). Moreover, the presence of other elements, such as Si, C, and O, was also confirmed.

**Figure 6:**
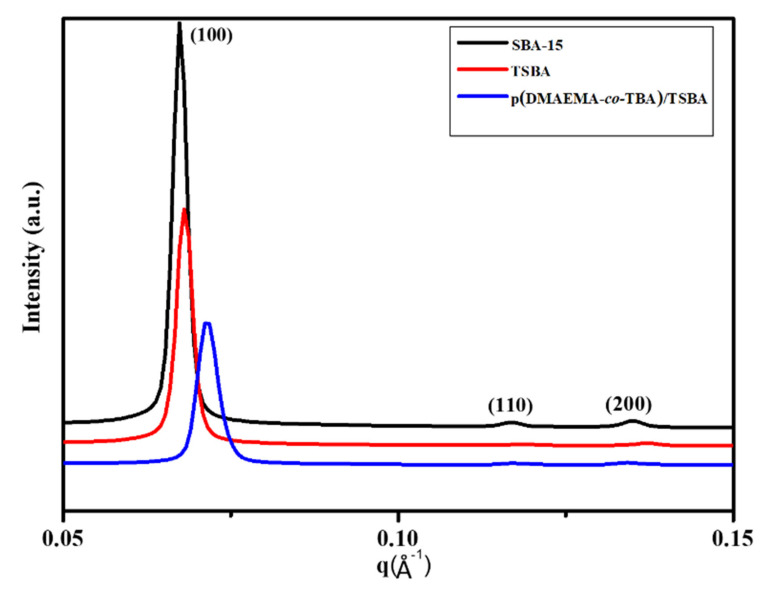
SAXS patterns of SBA-15, TSBA, and p(DMAEMA-*co*-TBA)/TSBA.

The small angle X-ray scattering (SAXS) patterns of materials are displayed in Figure 6. The SAXS patterns of pristine SBA-15 show three well-resolved peaks at a scattering vector, i.e., q* = 0.07, 0.12, and 0.14 Å^−1^, indexed to the (100), (110), and (220) reflection planes, respectively, which are associated with the 2D hexagonal pore arrangement of SBA-15. The structure of SBA-15 remained after the surface modification with TMSPM for TSBA, although a slight decrease in the intensity for the d spacing (110), (200), and (100) peaks was observed. The introduction of the copolymer to DMAEMA and TBA could slightly affect the mesoporous structure of SBA-15. After the grafting of the copolymer (i.e., p(DMAEMA-*co*-TBA)/TSBA), the intensities of the (110) and (200) peaks decreased, and the peak showing a maximum shifted slightly toward the right side, which demonstrates the diminishing of the ordered mesoporous structure due to the covering of the copolymer on the internal and external surface of TSBA, followed by a decrease in the pore size of the materials.

Wide angle XRD (WAXRD) patterns were recorded with the aim of confirming the presence of the crystalline CuNPs in the CuNPs/p(DMAEMA-*co*-TBA)/TSBA catalyst. Figure S1 illustrates the diffraction patterns of the catalyst. The catalyst exhibited typical diffraction patterns of CuNPs, with a small broad peak at approximately 2θ = 43°, 50°, and 73°, which corresponded to the (111), (200), and (220) crystal planes of CuNPs, respectively. The WAXRD pattern presents intense diffraction peaks in the range 2θ = 30–80°, representing monoclinic CuO. A broad diffraction peak of cuprite (111) was also observed at a diffraction angle of 36.2° (Figure S1). These diffraction peaks were similar in terms of angular positions to that of *fcc* pure bulk copper crystalline peaks, but were relatively broad [29,30]. We have calculated the particle size of CuNPs present in the CuNPs/p(DMAEMA-*co*-TBA)/TSBA catalyst and CuNPs/TSBA. The average particle size of the nanoparticles is around 1.8 nm for CuNPs/p(DMAEMA-*co*-TBA)/TSBA catalyst and CuNPs/TSBA, which is determined from the HRTEM image using the Image J software as shown in Figures S2 and S3 as well as from the Scherer’s equation [31]. The mean size of CuNPs estimated from Scherer’s equation was in good agreement with the data taken from the HRTEM image. In the case of CuNPs/SBA-15, however, the phases Cu_2_O and CuO were undetected. Peaks attributed to copper nanoparticles were not observed in the WAXRD pattern for CuNPs/SBA-15, which can be explained by the high dispersion of the copper oxide nanoparticles on the surface of SBA-15, which makes them undetectable by XRD pattern [30].

Figure S4 indicates the nitrogen adsorption–desorption isotherms for SBA-15, TSBA, and poly (DMAEMA-*co*-TBA)/TSBA, which exhibit typical type IV isotherms with H1 hysteresis loops as defined by IUPAC for all three materials, indicating that all three materials possess a mesoporous structure. The physico-chemical parameters of the samples are summarized in Table S1. The prepared SBA-15 had a Brunauer–Emmett–Teller (BET) surface area of 708 m^2^g^−1^, pore volume of 0.89 cm^3^g^−1^, and pore size of 7.7 nm. For functionalized SBA-15 (TSBA), the BET surface area, pore volume, and pore size were reduced to 600 m^2^g^−1^, 0.65 cm^3^g^−1^, and 6.8 nm, respectively. The variables agree with those reported in previous literature [27]. A higher BET surface area for the channels was observed in the SBA-15 samples. The existence of functional groups at the mesoporous surface promotes copolymerization on the surface. The results show that pore volume, pore size, and surface area are decreased after functionalization. This confirms that the functional group is located not only on the outer surface but also inside the mesoporous channel. After polymerization (i.e., p(DMAEMA-*co*-TBA)/TSBA), the surface area, pore volume, and pore size further decreased to 353 m^2^g^−1^, 0.46 cm^3^g^−1^, and 6.1 nm, respectively. The noticeable decrease in the physico-chemical parameters of the polymer-coated samples evidences the successful grafting of DMAEMA and TBA on the surface of SBA-15 [26,32].

**Figure 7:**
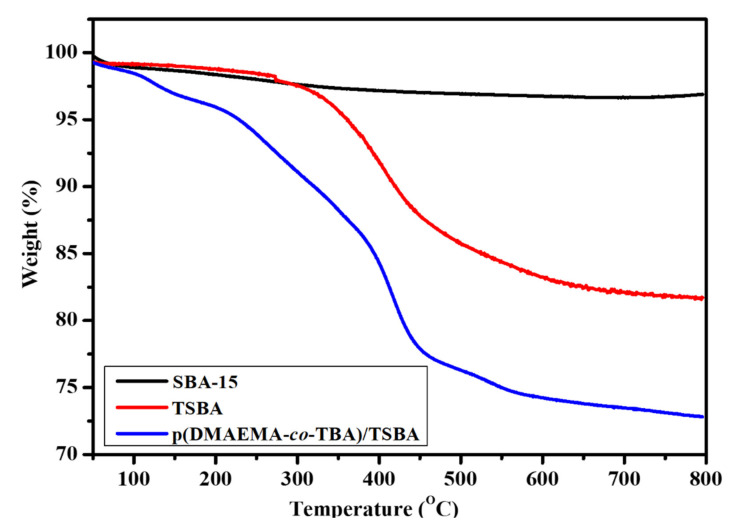
Thermogravimetric analysis (TGA) curves of SBA-15, TSBA, and p(DMAEMA-*co*-TBA)/TSBA.

In the thermogravimetric analysis (TGA) curves of Figure 7, the SBA-15, TSBA, and p(DMAEMA-*co*-TBA)/TSBA samples were heated from 25 °C to 800 °C at a rate of 10 °C/min under a N_2_ atmosphere. The main weight loss in the first region (below ca. 100 °C) originates from desorption of the physically adsorbed water on the surface. The mass loss was 3%, which can be considered a negligible value due to the very small rate of mass loss at the temperature. The calcined SBA-15 indicates complete removal of the surfactant during the calcination process, which proves the thermal stability of SBA-15. The weight loss up to 800 °C was found to be 15% after the surface modification of SBA-15 with TMSPM [26], whereas the weight loss at approximately 350–450 °C may be due to the degradation of TMSPM. Similarly, an additional 10% loss of the sample up to 800 °C, along with the weight loss at 200–350 °C, may be due to the decomposition of the copolymer, which indicates a reasonable amount of copolymer loading on the SBA-15 surface.

### 3.2. Stimuli-Responsive Performance

**Figure 8:**
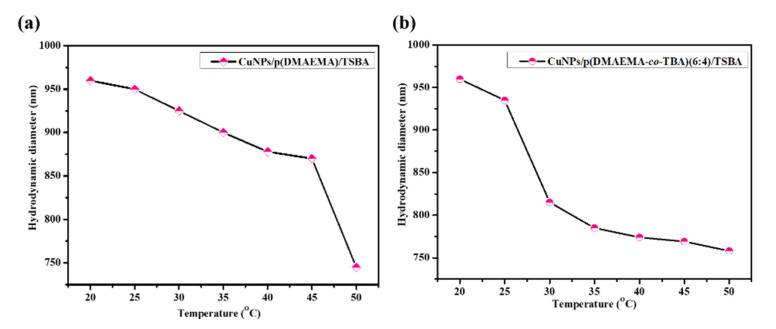
Particle size of (**a**) CuNPs/p(DMAEMA)/TSBA and (**b**) CuNPs/p(DMAEMA-*co*-TBA)(6:4)/TSBA as a function of temperature at pH = 7.

The temperature and pH dependent changes in solution behavior of the dual responsive copolymer grafted catalyst CuNPs/p(DMAEMA-*co*-TBA)/TSBA were studied using dynamic light scattering (DLS). We evaluated how the thermo-responsive monomer TBA influences the transition temperature or LCST of the copolymers, or how the presence of the hydrophobic co-monomer influences the self-assembly of the DMAEMA chains on mesoporous silica. To explore the temperature-responsive properties of the catalyst, the hydrodynamic diameters (D_H_) of the CuNPs/p(DMAEMA-*co*-TBA)/TSBA in aqueous solution at pH 7 at different temperatures were measured. Unless otherwise specified, p(DMAEMA-*co*-TBA)(6:4) was used throughout the text, where 6:4 stands for the comonomer ratio of DMAEMA to TBA, as described in the Section 2.1. The hydrodynamic diameter of CuNPs/p(DMAEMA-*co*-TBA)/TSB with different comonomer ratios was plotted as a function of temperature at pH 7 in Figure S5. In Figure 8a, the hydrodynamic diameter of CuNPs/p(DMAEMA)/TSBA as a function of temperature is given as a reference, where the comonomer ratio of DMAEA to TBA is 100:0.

The LCST of the CuNPs/p(DMAEMA-*co*-TBA)/TSBA decreased with increasing TBA content in the copolymer, as shown in Figure S5. For instance, the LCST of the CuNPs/p(DMAEMA-*co*-TBA)(6:4)/TSBA in aqueous solution was found to be in the range of 25–30 °C, whereas the CuNPs/p(DMAEMA)/TSBA particles displayed a fine transition in the range of 45–50 °C [27,33] (Figure 8 and Figure S5). Upon increasing the temperature from 20 to 50 °C, the hydrodynamic diameter of CuNPs/p(DMAEMA-*co*-TBA)(6:4)/TSBA particles noticeably decreased from 962 to 761 nm, which can be attributed to the hydrophobic interactions and hydrogen bonding. At low temperatures, the fragments arranged into a random coil conformation due to intermolecular hydrogen bonding interactions. As the temperature increased, the copolymer segments were dehydrated, and the intermolecular hydrophobic interactions dominated, resulting in the collapse of the copolymer segments on the surface of SBA-15. The LCST of p(DMAEMA) was lowered by copolymerization with a hydrophobic co-monomer (TBA). The LCST of the copolymer was found to be in the range of 25–30 °C. These results demonstrate the copolymerization of the TBA and DMAEMA monomers, which broadened and lowered the LCST [33,34]. This can be attributed to the fact that TBA content changes the molecule’s hydrophobicity. Obviously, the presence of the comonomer decreases the LCST, as previously reported. 

**Figure 9:**
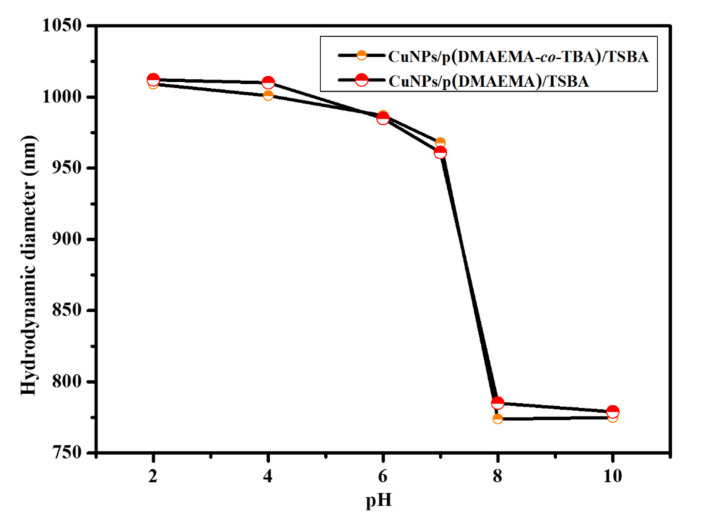
Hydrodynamic diameter of CuNPs/p(DMAEMA-*co*-TBA)/TSBA and CuNPs/p(DMAEMA)/TSBA at different pH values, as measured by dynamic light scattering (DLS).

The pH-responsive behavior of CuNPs/p(DMAEMA-*co*-TBA)/TSBA and CuNPs/p(DMAEMA)/TSBA was also investigated. The hydrodynamic diameter (D_H_) was measured at different pH values ranging from 2 to 10, as shown in Figure 9. At pH < 8, the polymer chains are protonated and reach a maximum swelling of 1110 nm. The hydration layer rises on the outer surfaces of SBA-15, which results in a larger particle diameter measurement. The changes in the hydrodynamic diameter of CuNPs/p(DMAEMA-*co*-TBA)/TSBA and CuNPs/p(DMAEMA)/TSBA at different pH values showed a pKa between 7 and 8, which agrees with previous literature [26]. Under acidic conditions, the polymer was stretched and in an opened state due to the protonation of tertiary amine functional groups in p(DMAEMA), owing to the extension of polymer chains. The repulsion between the positive charges formed on the amino groups (NH_3_^+^) was recognized on the copolymer with DMAEMA, which is responsible for the larger particle sizes observed at pH values of 2–6. Therefore, the reactants were able to diffuse into the pores of the nanocarriers. When the pH value increases, the amino groups are deprotonated (NH_2_), which results in an increase in the hydrophobic interaction; therefore, the D_H_ of the catalyst decreases due to deprotonation of the side ammonium group of the polymer. At pH 10, the compact polymer layer blocks the pores. This behavior was attributed to the increased hydrophilicity resulting from the ionization of the DMAEMA component taking place along the TBA chains [34].

### 3.3. Catalysis

#### 3.3.1. Optimization

To explore the catalytic behavior, the efficient reusable mesoporous silica-supported CuNPs/p(DMAEMA-*co*-TBA)/TSBA catalyst was activated by heating at 50 °C overnight and used for the oxidation of benzyl alcohol under mild conditions. The catalytic system was optimized using different variables: oxidants, temperature, solvent, and catalytic amounts. Initially, to evaluate the reaction conditions for the selective oxidation of alcohols, we used benzyl alcohol as a model substrate. 

Good conversions were achieved for dimethyl sulfoxide (DMSO), toluene, and water, but H_2_O was more eco-friendly than DMSO and toluene (Table S2). Among the oxidants, we found that H_2_O_2_ was more promising than other oxidizing agents, such as H_5_IO_6_ and *t*-BuOOH. In addition, it was found that 1.0 mmol of H_2_O_2_ was sufficient for maximum conversion (Table S3). Therefore, the activity of the CuNPs/p(DMAEMA-*co*-TBA)/TSBA catalyst for the oxidation of benzyl alcohols was investigated using H_2_O_2_ as oxidant throughout this work. In our future work, however, we will use molecular oxygen as oxidant because O_2_ is cheaper and an environmentally friendly oxidant than H_2_O_2_ to further reveal the catalytic activity of the present catalyst. 

It is also noteworthy that the conversion of benzaldehyde increased steadily with an increase of the catalyst amount. Furthermore, the catalytic conversion reached a maximum when 5 mg of catalyst was used. Increasing the amount of catalyst above 5 mg did not show any noticeable influence on the reactivity (Table S4).

The blank reaction was performed under identical conditions. The yield of benzaldehyde was only 8% in the control experiment without any catalyst or oxidant (Table S5, entry 12). In the absence of the oxidant, CuNPs/p(DMAEMA-*co*-TBA)/TSBA catalysts alone showed only a 41% yield of benzaldehyde (Table S3, entry 7), and in the absence of a catalyst, 18% (Table S5, entry 1) benzaldehyde was produced, indicating that H_2_O_2_ alone cannot oxidize benzyl alcohol into benzaldehyde. To increase the benzaldehyde yield, such promoters as oxidants and catalysts were added. When the reaction was carried out with pristine SBA-15 only (without CuNPs and copolymer), p(DMAEMA-*co*-TBA)/TSBA (without CuNPs), or CuNPs/p(DMAEMA-*co*-TBA) (without TSBA), 21%, 35%, 65%, conversion, respectively, of benzyl alcohol was achieved. This result hints that both the polymer and copper species must be efficient active sites for the reaction and that the presence of a suitable support is essential for a good catalysis (Table S5, entries 2, 8, 11). We conducted experiments using CuNPs/SBA-15 without a functionalized polymer, which resulted in moderate conversion (Table S5, entry 3). It should be noted here that if the actual loading of Cu for CuNPs/SBA-15 is lower than that of CuNPs/pDMAEMA-*co*-TBA)/TSBA, it cannot be said that the latter catalyst has higher catalytic activity than former one. As mentioned from the WAXRD pattern in Figure S1, however, the actual loadings of CuNPs of CuNPs/SBA-15 were hard to be estimated. Thus, even if direct comparison of the catalytic activities of these two catalysts may have some ambiguity, it is of no doubt the fact that the CuNPs/p(DMAEMA-*co*-TBA)/TSBA catalyst showed the highest activity with 99% of yield, whereas the CuNPs/SBA-15 did not improve the conversion (80%), which may be attributed to the synergetic effect of both polymer and CuNPs. The yield was significantly lower in the absence of CuNPs in the catalyst p(DMAEMA)/TSBA (Table S5, entry 4). To study the effect of the copolymer coating, the experiment was conducted with and without the TBA monomer at different temperatures (25 and 50 °C) in the presence of CuNPs (Table S5, entries 5 and 6). Interestingly, the results indicated the importance of TBA, with a slight increase in the conversion after copolymerization at room temperature. Even in the presence of CuNPs, p(DMAEMA) without TBA monomer showed low conversion (60%) (Table S5, entry 7). The influence of other reaction conditions on the catalytic performance was investigated. 

The temperature-responsive environment of the catalyst was predicted from the DLS measurements. Then, we carried out the reaction under different temperature conditions, i.e., above and below the LCST. The LCST of CuNPs/p(DMAEMA)/TSBA was 45–50 °C. After copolymerization between DMAEMA and TBA on SBA-15, the LCST value decreased to 25–30 °C. Therefore, we tested the oxidation reaction at room temperature (below LCST) and 50 °C (above LCST), and found that benzyl alcohol was converted to benzaldehyde at rates of 99% and 91%, respectively (Table S5, entries 9 and 10). This provides clear evidence that the shrinking property was much higher for the catalyst at 50 °C. On shrinking, the polymer-coated CuNPs/p(DMAEMA-*co*-TBA)/TSBA behaves like a wall and obstructs the outgoing of reactants to the metal surface inside the pores, which negatively affects the reaction rate. Below the LCST, the polymer enlarges and opens the pores of mesoporous silica SBA-15, which leads to better interaction between the active sites of the CuNPs inside the pores and the reactants facing, resulting in better organic conversion. DLS studies showed that the catalyst swelled at acidic pH. The oxidation reaction of benzyl alcohol was investigated under different pH conditions to study the pH-responsive behavior of the catalyst. The repulsion between the positive charges in NH_3_^+^ was large, and the swelling property was enhanced at low pH, which resulted in better conversion (99%) (Table S5, entry 10).

#### 3.3.2. Extension of Scope

The scope of the CuNPs/p(DMAEMA-*co-*TBA)/TSBA-catalyzed oxidation of various substituted alcohols was examined (Table 1). The results indicate that aromatic alcohols usually require less reaction time and result in good conversion compared to aliphatic alcohols due to the good interaction between the aromatic alcohols and catalyst surface through π–π bonding [35,36]. The active oxidation of benzyl alcohol is due to the active phenyl group. Benzaldehyde was the main oxidation product in this system with 99% conversion and 99% selectivity (Table 1, entry 1). Various mono-substituted benzyl alcohols were utilized to produce aldehydes and ketones in high yields (Table 1, entries 1–15). In certain catalytic systems, it was not easy to obtain the desired benzaldehyde in the oxidation of benzyl alcohol, but benzoic acid was often produced instead of benzaldehyde. Ronzer et al., reported Na_2_[WZnZn_2_(H_2_O)_2_(ZnW_9_O_34_)_2_ catalyst that oxidized primary alcohols to the benzyl alcohol as well as other corresponding carboxylic acids [37].

*Ortho*-substituted benzyl alcohols produced the desired aldehydes (Table 1, entries 2 and 3) in lower yields, in part due to steric hindrance. Additionally, a heterocyclic system could achieve better conversion than aromatic substituted alcohols with good selectivity (Table 1, entry 5). The decrease in the yield was caused by the substitution of strong electron-withdrawing nitro and OH substituents (Table 1, entries 6 and 7), whereas halogen substituents produced a modest yield (Table 1, entry 2). 

Unfortunately the non-benzylic primary alcohols such as 1-butanol and 1-hexanol gave less conversion than that of the non-benzylic alcohols with more alkyl chains (Table 1, entries 8 and 9). The rate of conversion increased with the increasing chain length (Table 1, entries 10 and 11). When the catalyst was used in an allyl alcohol oxidation, the desired α, β-unsaturated aldehyde was produced with a 32% yield (Table 1, entry 12). To expand the substrate scope, the oxidation of cyclic alcohols, such as cyclohexanol and cyclopentanol, was also tested, which are also quantitatively converted to their corresponding aldehydes and ketones with very good yields (Table 1, entries 13 and 14). All the corresponding aldehydes were produced with high selectivity, and over-oxidation into carboxylic acid was not observed [39]. ^1^H/^13^C NMR spectra of oxidized products are included in the Supplementary Data (Figure S6). Turnover number (TON) and turnover frequency (TOF) values are also included in Table 1. TOF ranged from 1 to 42 for the oxidation of alcohols under aqueous conditions. 

To demonstrate the advantages of CuNPs/p(DMAEMA-*co-*TBA)/TSBA as a heterogeneous catalyst in this reaction, our results were compared with other homogeneous and heterogeneous catalysis of this reaction in the literature [27] (Table 2). TON and TOF values are also included in Table 2 for better comparison of catalytic activity of various catalysts. TOF ranged from 2 to 460 for the oxidation of benzyl alcohols under various reaction conditions. The TON and TOF values of the CuNPs/p(DMAEMA-*co*-TBA)/TSBA catalyst were found to be comparable with previous reported literatures. The results show that our catalyst is superior to or at least comparable with other catalysts in terms of the yields and reaction times.

### 3.4. Reusability of the Catalyst

**Figure 10:**
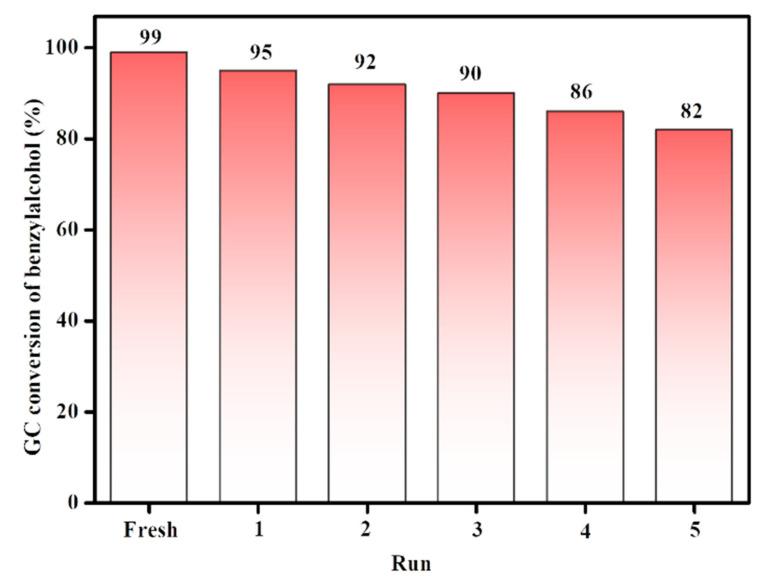
Recyclability of CuNPs/p(DMAEMA-*co*-TBA)/TSBA hybrid catalyst on the oxidation of alcohols.

One of the major reasons for supporting metal nanoparticles incorporated onto polymer-coated mesoporous SBA-15 is to enhance the activity of the catalyst. In addition to reaction conditions such as reaction temperature and time, recyclability is also crucial for evaluating catalysts. For this, recycling experiments were performed on the CuNPs/p(DMAEMA-*co-*TBA)/TSBA catalyst. To study the stability and reusability of the heterogeneous catalysts, they were recovered through centrifugation, washed with ethanol, dried at 50 °C for 5 h, and used in the recycling study. The yield and structure of the final products were compared with those tested for the fresh catalyst. The obtained results of the catalysts indicate that the catalytic activity decreased after recycling (Figure 10). It may be assumed that the slight deactivation of the catalyst after recycling might be due to the loss of some catalyst during recycling test as well as the oxidation of fouling or aging of the catalyst. For instance, hydroxyl radicals may damage the polymeric structure on the surface of the hybrid materials after the fifth wash, which may potentially impact the catalytic performance. In addition, metal leaching may take place during continuous washing and recycling for further use. 

WAXRD patterns indicate that there were no changes in the crystallinity of the CuNPs after the oxidation reaction, as shown in Figure S7. FETEM analysis clarifies the presence of CuNPs both inside and outside the pores. The black spots indicate the presence of CuNPs (Figure S8). FT-IR analysis was also conducted after the fifth cycle of the catalyst to investigate the presence of functional groups (Figure S9). Two weak peaks observed at 2916 and 2848 cm^−1^ prove the existence of C-H stretching, which was associated with the aliphatic group. A strong peak at 1728 cm^−1^, corresponding to the C=O group confirms that the catalyst structure was retained after the synthesis of aldehyde during the reaction. It is interesting to note that the CuNPs/p(DMAEMA-*co-*TBA)/TSBA catalyst for this work was found to be more cost-effective for preparation than simple metal nanoparticles such as CuNPs and FeNPs by our rough estimation, as shown in Tables S6–S9.

Before concluding, it should be noted that studies on the degradation mechanism and kinetics are quite needed to understand the catalytic activity for oxidation of various alcohols in depth. According to previous reports, for instance, the reaction of H_2_O_2_ with CuNPs under acidic or neutral conditions generates more hydroxyl radicles, which will increase the rate of the oxidation reaction [49,50]. In this work, the pH of the reacting mixture was about 7, which may enhance the catalytic activity. Thus further works on the subject should be conducted in order to interpret the results in this work in more depth for our future work. In this sense, our future work will include the degradation kinetics, i.e., the removal rate as a function of time, which is a key information to compare with other catalysts, as well as the degradation mechanism by the verification of radical species with the typical scavenger results, so that we could reveal whether hydroxyl radical is the main oxidant for alcohols degradation or not, etc.

## 4. Conclusions

In this study, CuNPs were successfully immobilized on a temperature- and pH-responsive copolymer with mesoporous silica SBA-15, which was found to be a highly active, eco-friendly, and recyclable catalyst for the selective oxidation of alcohols in water at room temperature. Under optimized reaction conditions, the catalyst was highly effective for the oxidation of various alcohols with substituted alkyl, aryl, and heterocyclic alcohols in the aqueous media using H_2_O_2_ as an oxidant at room temperature. The CuNPs/p(DMAEMA-*co-*TBA)/TSBA catalyst exhibited some responsive behavior at a certain temperature and pH. The lower critical solution temperature (LCST) became lowered by co-polymerization with a hydrophobic co-monomer (TBA). The LCST of the copolymer was found to be in the range of 25–30 °C. At low temperature and acidic environment, the benzyl alcohol conversion was found to be high. At high temperatures under basic conditions, however, the yield of conversion was less due to the swelling/shrinking of the copolymer coated on mesoporous silica SBA-15. In addition, the catalyst can be reused for at least five reaction cycles without significant loss of catalytic activity. In summary, the hybrid catalyst developed in this study exhibited outstanding alcohol conversion (up to 99%) within a short reaction time (1 h). It should also be noted that only a small amount of catalyst (5 mg) is needed to secure such an efficient catalytic activity. 

## Figures and Tables

**Table 1:**
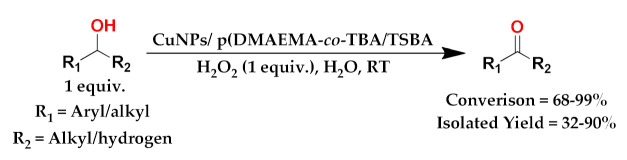
Oxidation of alcohols using copper nanoparticles (CuNPs)/p(DMAEMA-*co*-TBA)/TSBA catalyst ^a^.

Entry	Substrate	Product	Time (min)	Conversion ^b^ (Selectivity ^c^) (%)	Isolated Yield ^d^ (%)	TON ^d^	TOF ^d^
1	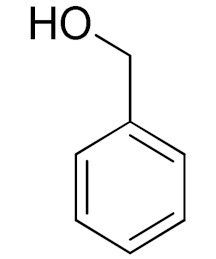	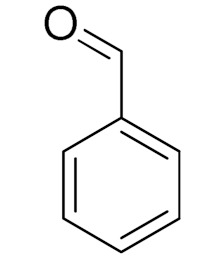	45	99 (99)	90	31	42
2	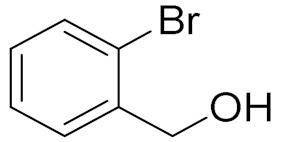	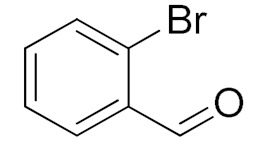	180	82 (98)	71	26	9
3	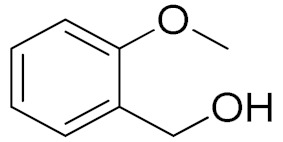	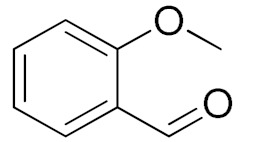	180	80 (94)	74	25	9
4	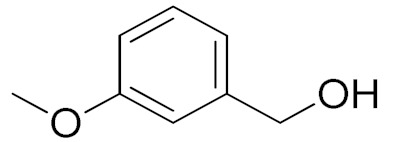	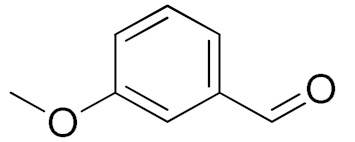	240	88 (98)	78	28	7
5	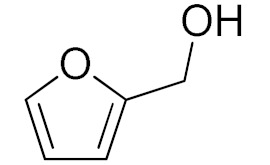	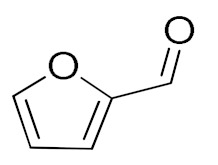	120	94 (99)	87	30	15
6	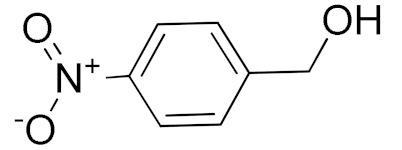	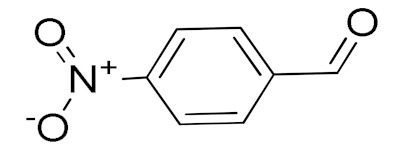	300	87 (95)	79	28	6
7	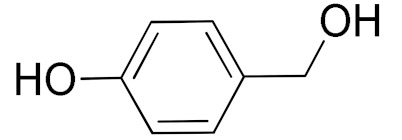	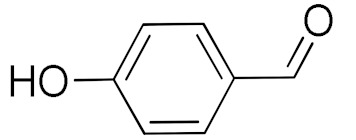	480	89 (99)	80	28	4
8	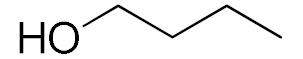	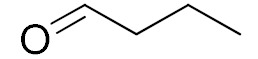	1440	76 (97)	62	24	1
9	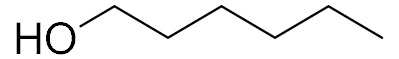	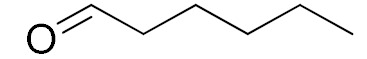	1440	75 (89)	88	24	1
10	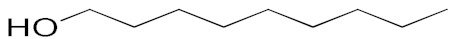	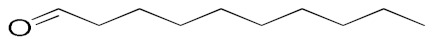	720	88 (97)	81	28	3
11	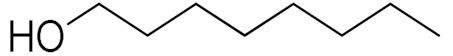	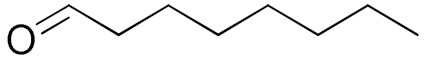	720	89 (88)	70	28	3
12	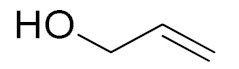	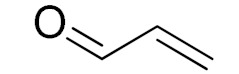	180	68 (88)	32	22	7
13	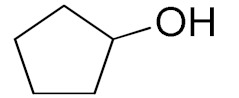	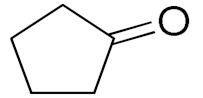	300	60 (95)	52	19	4
14	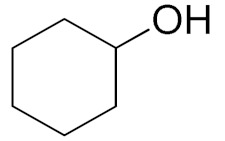	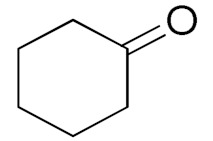	300	62 (91)	54	20	4
15	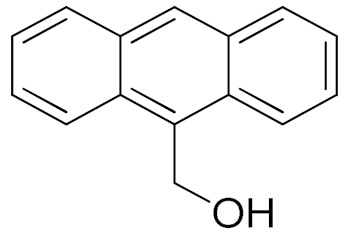	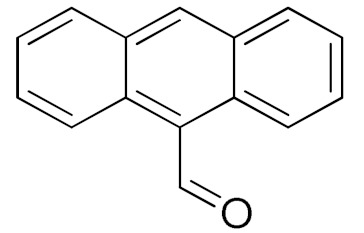	1440	91 (99)	83	29	2

^a^ Reaction conditions: Substrate (1 mmol), catalyst (5.0 mg), H_2_O_2_ (1 mmol), water (5 mL), 25 °C. ^b^ Determined by GC. ^c^ Selectivity in the parentheses were calculated from GC. ^d^ Turnover number (TON) and turnover frequency (TOF) were calculated according to the reference [38].

**Table 2:** Comparison of catalytic activities of the oxidation of benzyl alcohol for catalysts reported in literature and this work.

Entry	Catalyst	Oxidant	Catalyst Amount (mg)	Time (h)	Temp. (°C)	Yield (%)	TON	TOF	Ref.
1	Cu(NO_3_)_2_	H_2_O_2_ (1.2 mL)	10	1.5	80	52	78	52	[40]
2	Pd@Cu(II)-MOF	O_2_	23	25	130	99	50	2	[41]
3	CuSO_4_	H_2_O_2_ (1 mL)	10	1.5	80	62	186	124	[42]
4	CuCl_2_/CH_3_COCH_3_	O_2_	25	1	25	95	190	190	[43]
5	CuMn_2_ oxide	O_2_	200	0.6	102	99	-	-	[44]
6	CuCl_2_/THF	O_2_	20	2	80	85	43	22	[45]
7	CuSH/1NaK	*t-*BuOOH (1.6 mL)	20	24	70	72	165	7	[46]
8	Au-Cu/SiO_2_	O_2_	200	10	319	92	702	280	[47]
9	AgCu/SiC	O_2_	280	2.5	280	99	1151	460	[48]
10	CuNPs/p(DMAEMA-*co*-TBA)/TSBA	H_2_O_2_ (1 mL)	5.0	1	25	99	31	42	This work

Note: Cu(NO_3_)_2_: copper nitrate catalyst with the presence of H_2_O_2_ oxidant, without adding any additive. Pd@Cu (II)-MOF: The metal-organic zeolite imidazolate framework-8 supported palladium-copper bimetallic catalysts with a core-shell structure. CuSO_4_: copper sulphate catalyst with the presence of H_2_O_2_ oxidant, without adding any additive. CuCl_2_/CH_3_COCH_3_: Photo catalytic oxidation of benzyl alcohol by homogeneous CuCl_2_, CuMn_2_ oxide: copper-manganese mixed oxide nanoparticles prepared by co-precipitation method for selective oxidation of benzyl alcohol using molecular oxygen as an oxidant, CuCl_2_/THF: The catalytic system consists of CuCl_2_ and 2,2′-biquiniline 4-4′ dicarboxylic acid dipotassium salt (BQC) and was used for selective oxidation, CuSH/1NaK: oxidation in the presence of the crystalline copper silicates CuSH-1NaK, *t*-BuOOH oxidant and acetonitrile solvent, Au-Cu/SiO_2_: This bimetallic gold-copper on silica catalyst was prepared in a simple impregnation, AgCu/SiC: The synergistic effect between silver and oxide of Cu_2_O is explained and is supported with silicon carbide, CuNPs/p(DMAEMA-*co*-TBA)/TSBA: Copper nanoparticle decorated on copolymerized SBA-15 mesoporous silica material.

## Supplementary Materials

The following are available online at https://www.mdpi.com/2079-4991/10/10/2051/s1, Chemicals, Preparation of SBA-15 and SBA-15 functionalized with TMSPM, Materials characterization, Figure S1: Wide angle XRD (WAXRD) patterns of (a) copper nanoparticles (CuNPs)/SBA-15 and (b) CuNPs/p(DMAEMA-*co*-TBA)/TSBA; Figure S2: (a) High-resolution transmission electron microscopy (HRTEM) image of CuNPs/SBA-15 catalyst, and (b) particle size histograms of CuNPs; Figure S3: (a) HRTEM image of CuNPs/p(DMAEMA-*co*-TBA)/TSBA catalyst. (b) Particle size histograms of CuNPs; Figure S4: N_2_ adsorption-desorption isotherms and pore size distribution curves (inset) of (a) SBA-15, (b) TSBA, and (c) p(DMAEMA-*co*-TBA)/TSBA; Figure S5: Particle size of CuNPs/p(DMAEMA-*co*-TBA)/TSB at different monomer ratios of (a) 9:1, (b) 8:2, (c) 7:3, and (d) 6:4 plotted as a function of temperature at pH = 7; Figure S6: ^1^H and ^13^C NMR spectra of products; Figure S7: WAXRD patterns of CuNPs/p(DMAEMA-*co*-TBA)/TSBA hybrid catalyst on the oxidation of alcohol after fifth wash; Figure S8: HRTEM image of CuNPs/p(DMAEMA-*co*-TBA)/TSBA hybrid catalyst on the oxidation of alcohol after fifth wash; Figure S9: FT-IR spectra of CuNPs/p(DMAEMA-*co*-TBA)/TSBA hybrid catalyst on the oxidation of alcohols at (a) fresh and (b) after fifth wash; Table S1: Brunauer–Emmett–Teller (BET) surface area, pore volume, and pore sizes distribution values of SBA 15, TSBA, and p(DMAEMA-*co*-TBA)/TSBA; Table S2: Effect of solvent in oxidation of benzyl alcohol; Table S3: Effect of oxidants in oxidation of benzyl alcohol; Table S4: Effect of catalyst amount in oxidation of benzyl alcohol; Table S5: Effect of different catalysts in oxidation of benzyl alcohol; Table S6: Total expense for the synthesis of CuNPs/p(DMAEMA-*co*-TBA)/TSBA hybrid catalyst; Table S7: Total expense for the synthesis of Copper nanoparticles; Table S8: Total expense for the Synthesis of Iron nanoparticles; Table S9: Total expense for the Synthesis of Iron nanoparticles.
