# Supplementary material for: Dual Stimuli-Responsive Copper Nanoparticles Decorated SBA-15: A Highly Efficient Catalyst for the Oxidation of Alcohols in Water
Source: Nanomaterials (Basel). 2020 Oct 16;10(10):2051. doi: 10.3390/nano10102051 (PMC7603010; doi:10.3390/nano10102051)
Supplement: Supplementary file 1 [file nanomaterials-10-02051-s001.pdf]

nanomaterials

Supplementary Materials

Dual Stimuli-Responsive Copper Nanoparticles Decorated SBA-15: A Highly Efficient Catalyst for the Oxidation of Alcohols in Water

Anju Maria Thomas, Jerome Peter, Saravanan Nagappan, Anandhu Mohan and Chang-Sik Ha*

Department of Polymer Science and Engineering, Pusan National University, Busan 46241, Korea; anju.05u0@gmail.com (A.M.T.); jestro.12@gmail.com (J.P.); saravananagappa@gmail.com (S.N.); anandhumohangcm@gmail.com (A.M.)

* Correspondence: csha@pnu.edu; Tel.: +82-51-510-2407

Chemicals

Tetraethyl orthosilicate (TEOS) ($(C_2H_5O)_4Si$), Pluronic (poly(ethylene glycol)-block-poly(propylene glycol)-block-poly(ethylene glycol)) (P123), toluene ($C_6H_5CH_3$), hydrochloric acid (HCl), 3-trimethoxysilylpropyl methacrylate (TMM), dimethyl aminoethyl methacrylate (DMAEMA), *tert*-butyl acrylamide (TBA), copper(II) bromide, potassium persulfate ($K_2S_2O_8$), sodium borohydride ($NaBH_4$), benzyl alcohol, hydrogen peroxide (H_2O_2), periodic acid (H_5IO_6), potassium tert-butoxide (*t*-BuOK), Ethanol, methanol, diethyl ether, and ethyl acetate, respectively were purchased from Sigma-Aldrich company. Glassware used in the experimental work was washed with distilled water and dried at 100 °C overnight. All the chemicals employed for the synthesis were of analytical grade and used as commercially available without any further purification.

Preparation of SBA-15

SBA-15 was prepared according to our previous study [1]. During a typical synthesis procedure 16 g of Pluronic (P123) block copolymer was dissolved in 500 mL deionized water and 80 mL HCl (35%) solution. The mixture was vigorously stirred for 6 h at room temperature to attain complete dissolution of block copolymer. When the solution become fully transparent, 36.8 mL TEOS was added dropwise to the homogeneous solution while stirring, then kept at 35 °C for 24 h under a static condition. Following this, the mixture was aged at 100 °C for 24 h. The white solid product formed was recovered by filtration and washed with water and ethanol to remove surfactants. The resulting products were air dried and calcined at 550 °C for 8 h to remove the template.

Preparation of SBA-15 functionalised with TMSPM

The typical silylation of the mesoporous SBA-15 silica was done according to the procedure reported in the literature [1]. 1 g of mesoporous SBA-15 and 3 mL trimethoxy silyl propyl methacrylate (TMSPM) were added into a three-neck flask containing 60 mL of toluene. The flask was then equipped with a water condenser and nitrogen atmosphere and heated at 110 °C for 24 h. The modified SBA-15 was separated by centrifugation, then washed in toluene, dichloromethane, and water, and finally the obtained product was dried in a vacuum for 12 h to remove solvent traces. The obtained product was named TSBA.

Materials characterisation

XRD patterns of the samples were achieved by a refractometer (35 kV, 28.5 mA, and 298 K; MiniFlex X-ray diffractometer). The Fourier transform infrared (FT-IR JASCO, FTIR 4100) spectrometer used a transmission mode ranging from 400 to 4000 cm^{-1} to identify functional groups. The nitrogen adsorption–desorption isotherms at 77 K were obtained using a surface area and pore size analyser (Micromeritics ASAP 2020 V3.04 G). The specific surfaces area was calculated by using the standard Brunauer–Emmett–Teller (BET) analysis. The pore parameters were analysed from the

desorption branch of these isotherms using Barrett–Joyner–Halenda (BJH) analysis. Field emission scanning electron microscopy (FE-SEM, SUPRA-25, 20 Kv) images of dried up mesoporous SBA-15 particles were acquired after the samples were sputter-coated with platinum. Transmission electron microscopy (HRTEM, TEM-2011) images were collected at an accelerating voltage of 200 kV affixed with an energy-dispersive X-ray (EDX) spectrometer. The elemental mapping was performed with a high-angle annular dark-field (HAADF) detector and Talos F200X system. The examined samples dispersed in ethanol were made by depositing a drop of the SBA-15 suspension on a copper grid. A VGESCALAB 220-IXL spectrometer was used to measure X-ray photoelectron spectra (XPS) with an Alka X-ray source (1486.6 eV). Thermogravimetric analysis (TGA, Q50 V6.2, Build 187, TA instruments, USA) was done under N_2 flow and the samples were heated from 30 to 800 °C to evaluate thermal stability and organic substances on the modified and polymerized SBA-15. ^{1}H and ^{13}C nuclear magnetic resonance (NMR) spectra were obtained on Bruker (500 MHz) spectrometer with DMSO as a solvent.

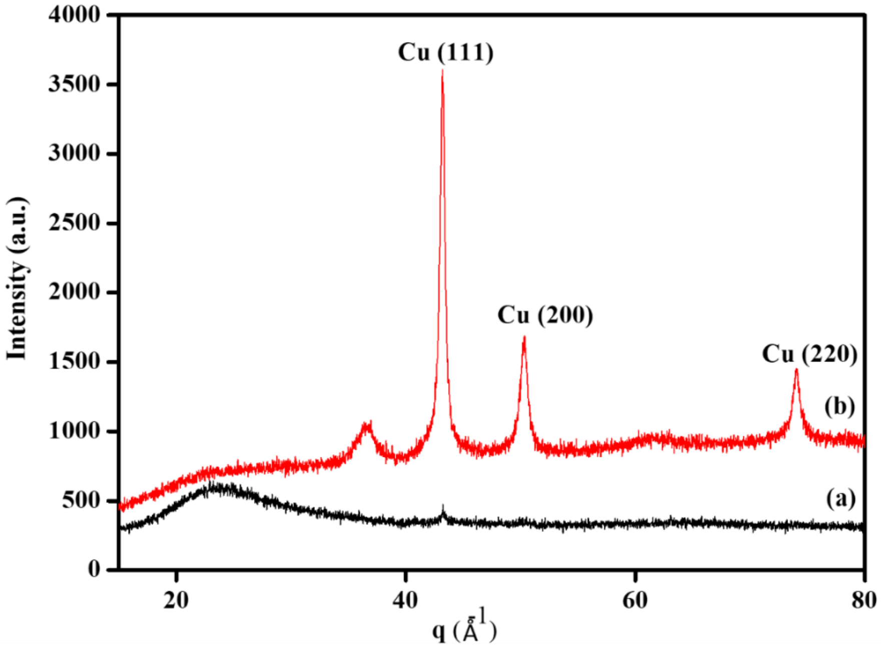

Figure S1. Wide angle XRD (WAXRD) patterns of (**a**) copper nanoparticles (CuNPs)/SBA-15 and (**b**) CuNPs/p(DMAEMA-*co*-TBA)/TSBA.

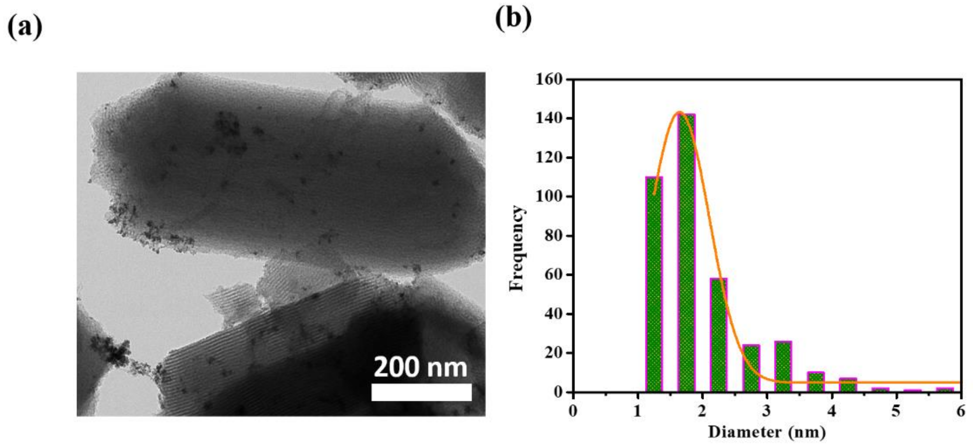

Figure S2. (**a**) High-resolution transmission electron microscopy (HRTEM) image of CuNPs/SBA-15 catalyst, and (**b**) particle size histograms of CuNPs.

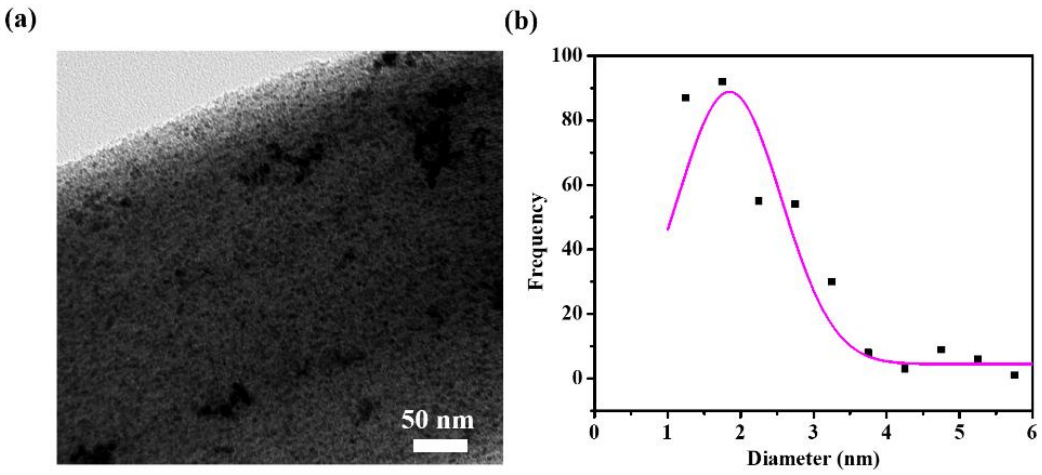

Figure S3. (**a**) HRTEM image of CuNPs/p(DMAEMA-*co*-TBA)/TSBA catalyst. (**b**) Particle size histograms of CuNPs.

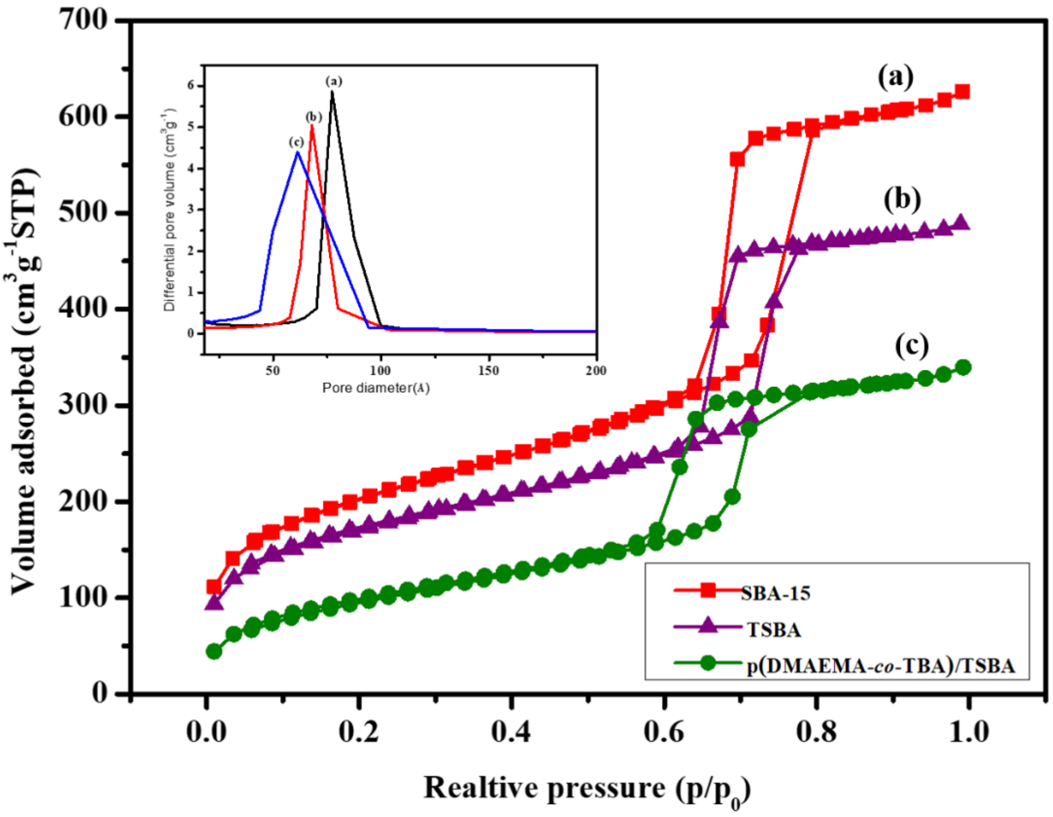

Figure S4. N_2 adsorption-desorption isotherms and pore size distribution curves (inset) of (**a**) SBA-15, (**b**) TSBA, and (**c**) p(DMAEMA-*co*-TBA)/TSBA.

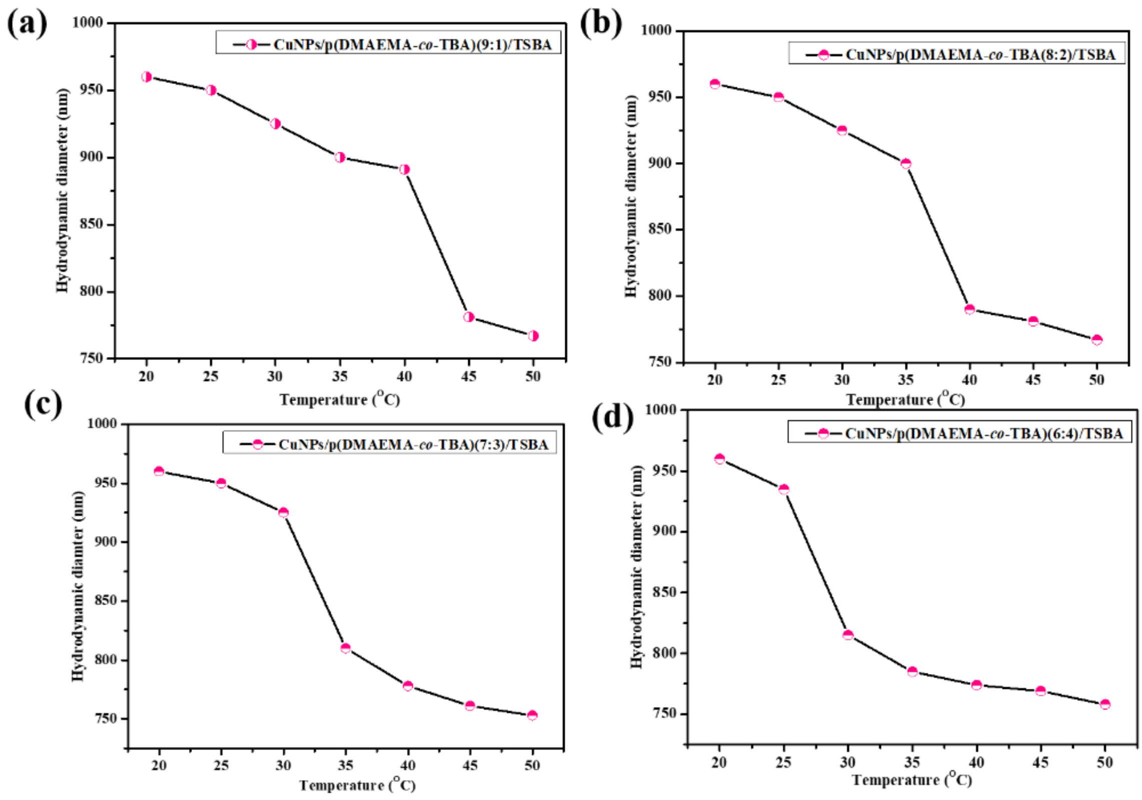

Figure S5. Particle size of CuNPs/p(DMAEMA-*co*-TBA)/TSB at different monomer ratios of (**a**) 9:1, (**b**) 8:2, (**c**) 7:3, and (**d**) 6:4 plotted as a function of temperature at pH = 7.

Benzaldehyde (^{1}H NMR spectrum)

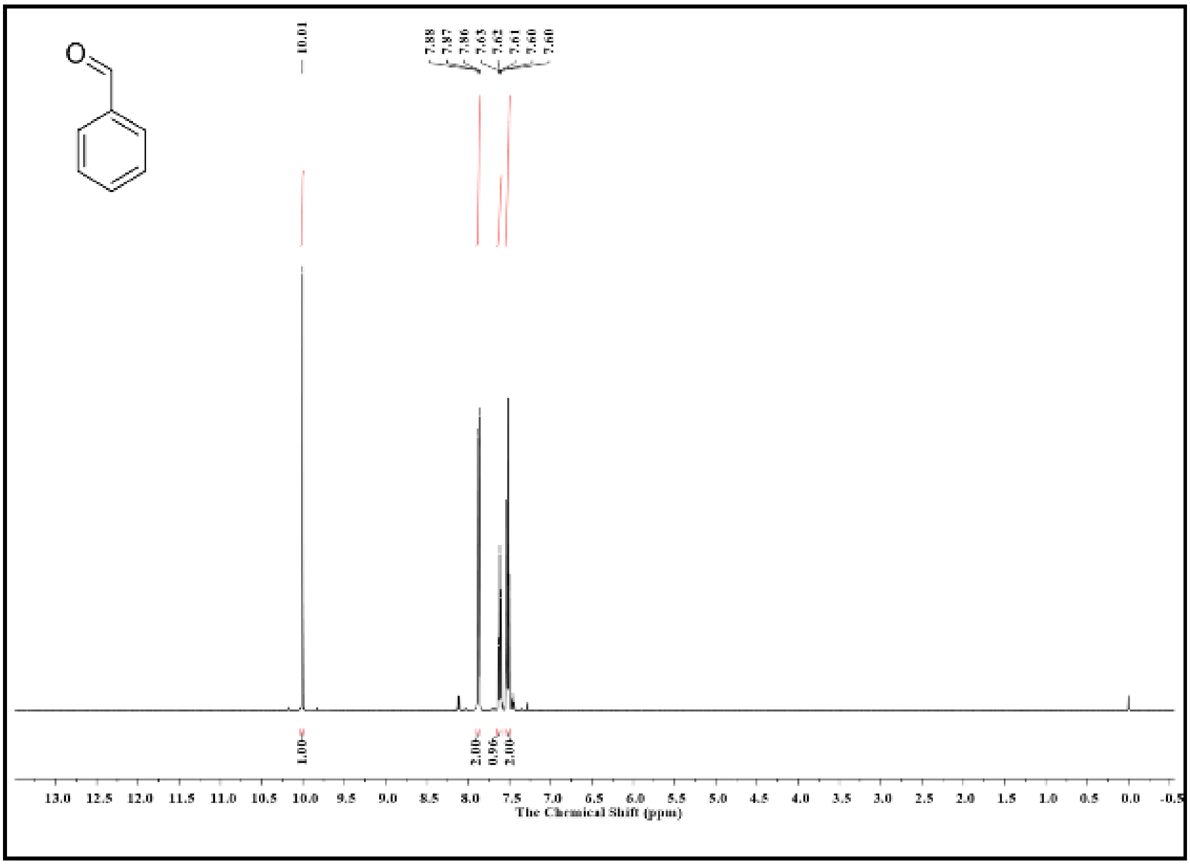

Benzaldehyde (^{13}C NMR spectrum)

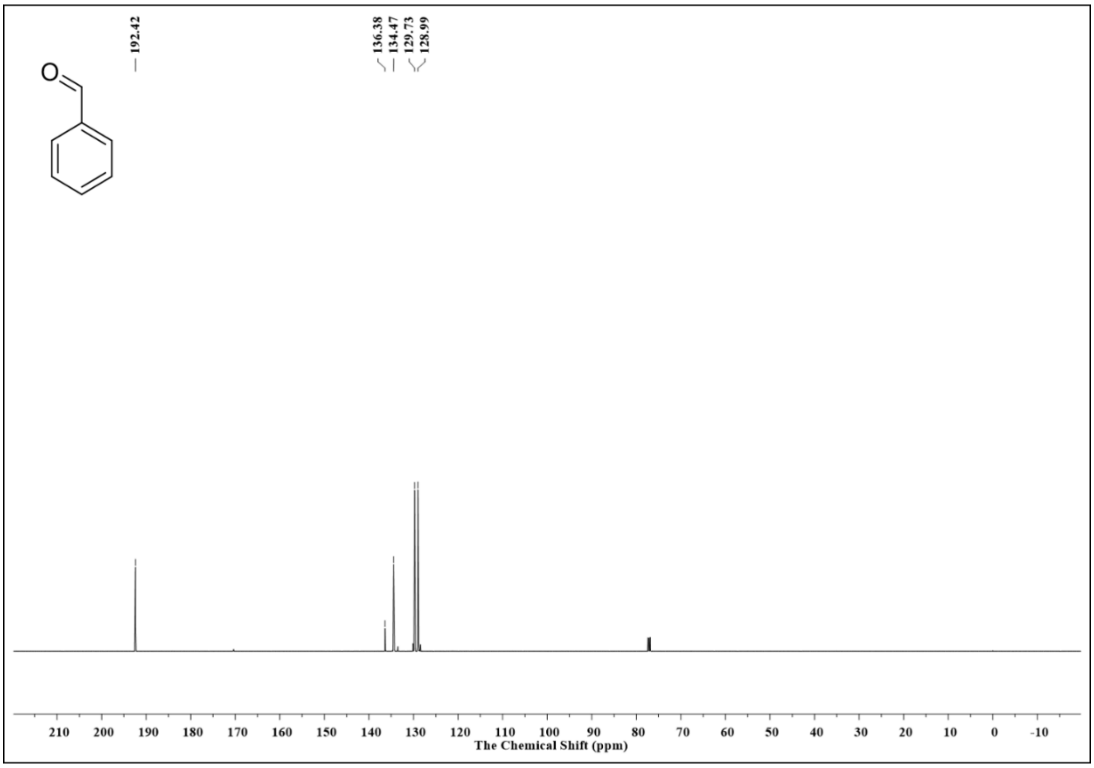

Cyclopentanone (^{1}H NMR spectrum)

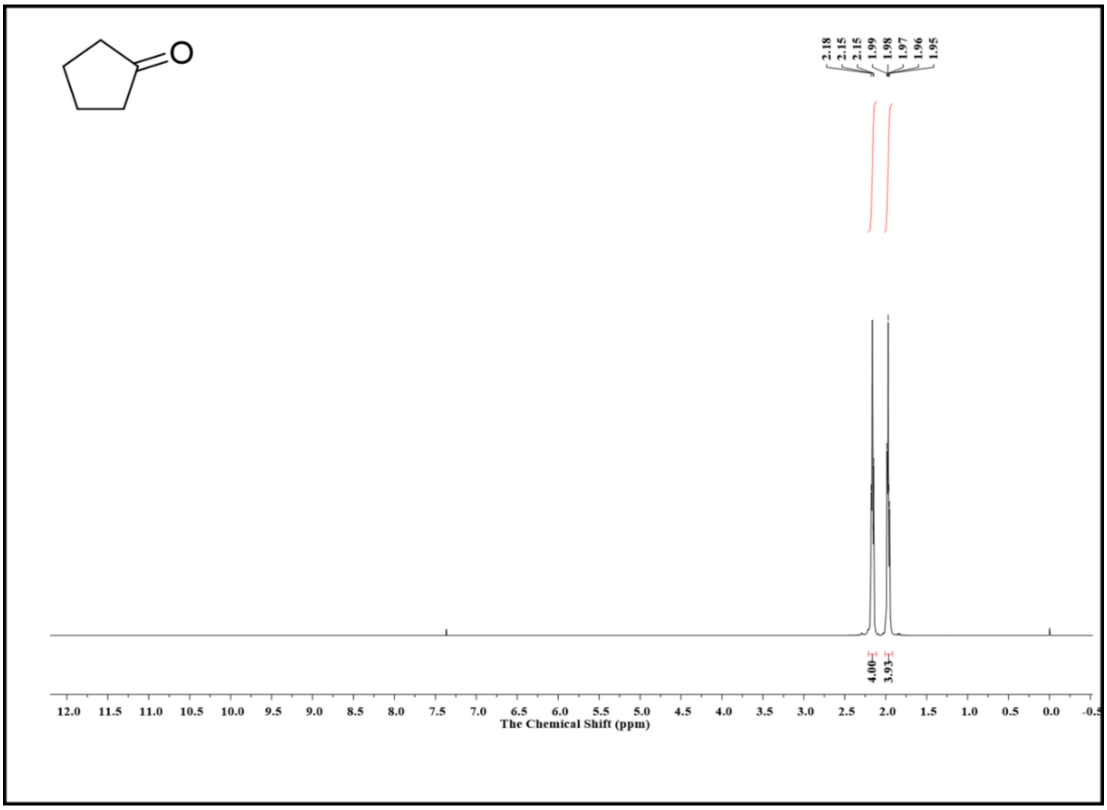

Cyclopentanone (^{13}C NMR spectrum)

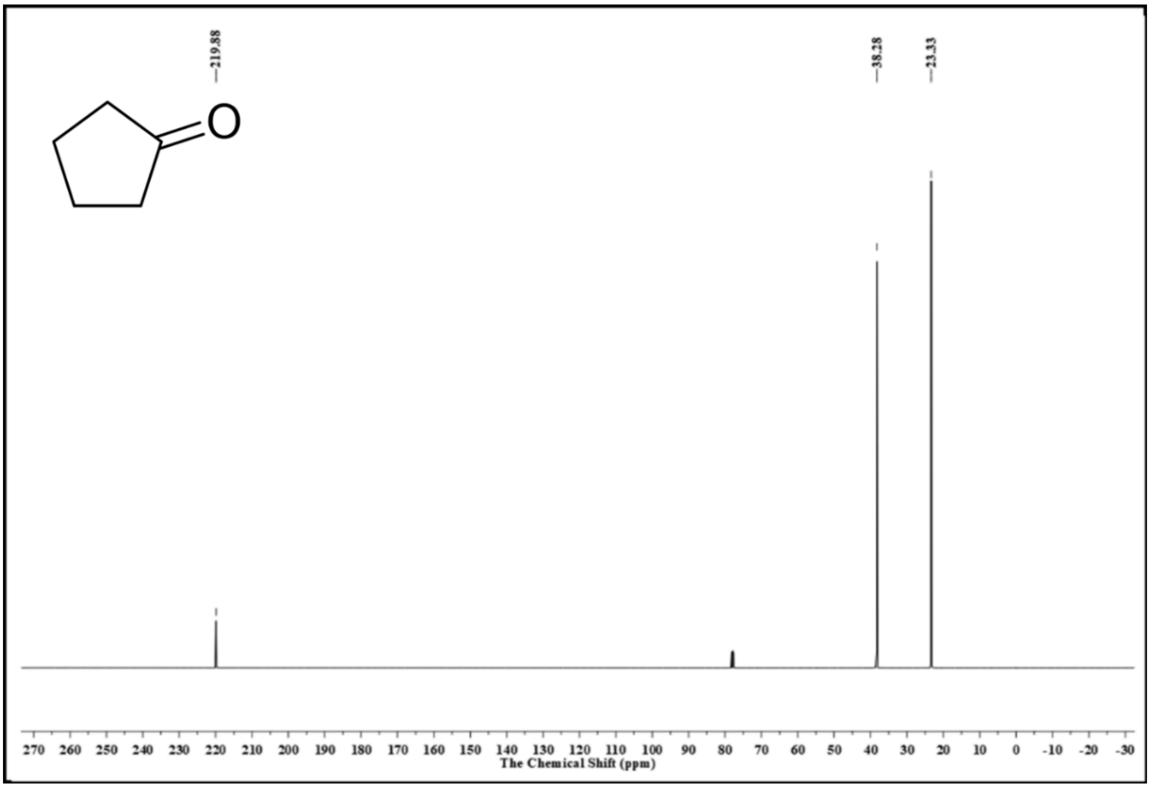

2-Methoxy benzaldehyde (^{1}H NMR spectrum)

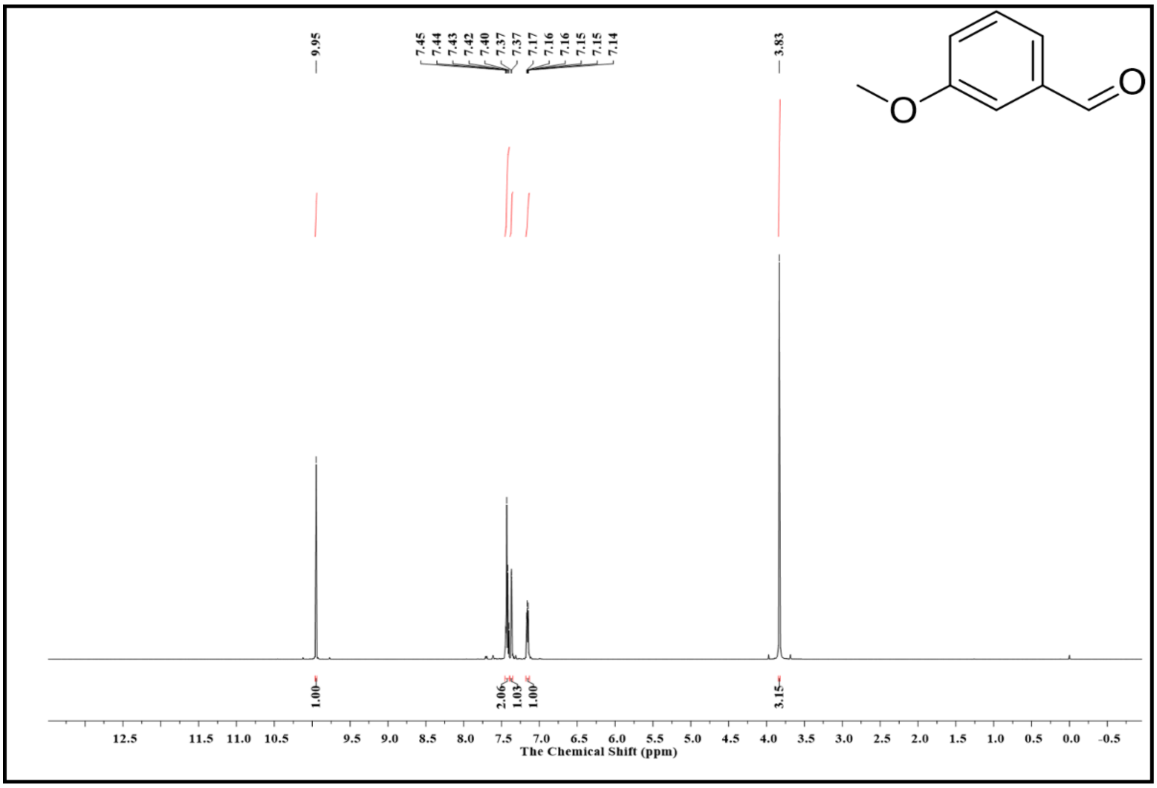

2-Methoxy benzaldehyde (^{13}C NMR spectrum)

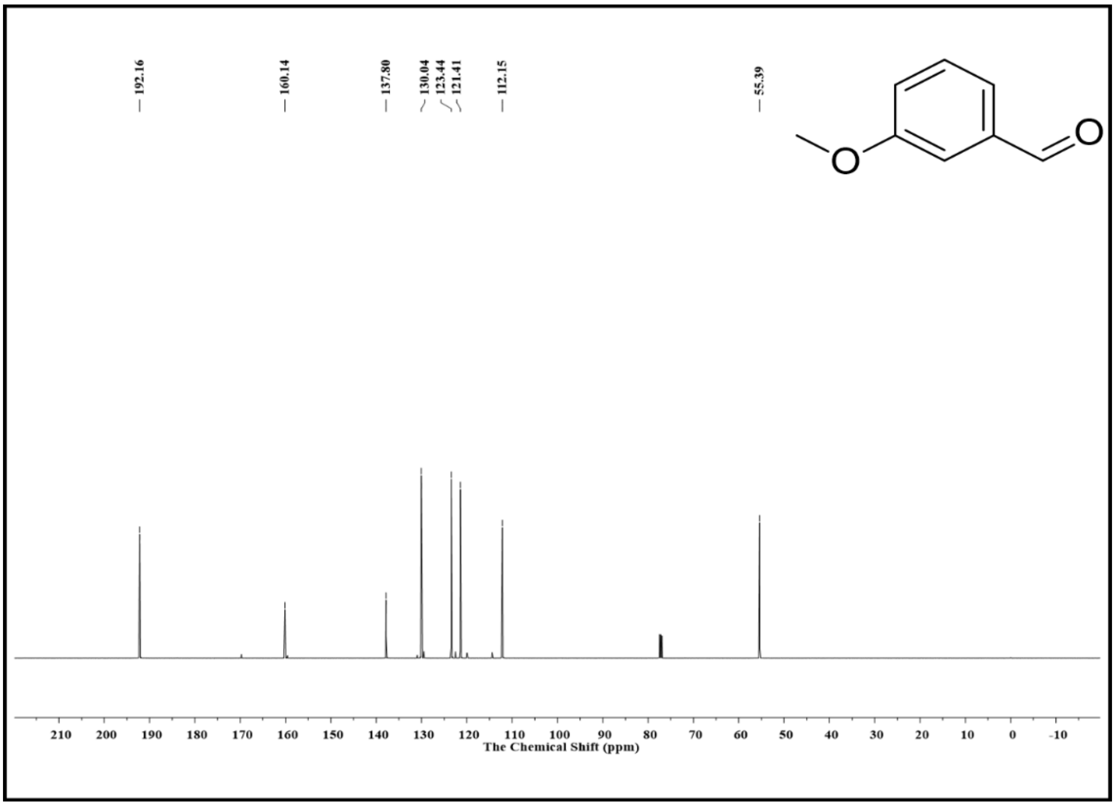

3-Bromobenzaldehyde (^{1}H NMR spectrum)

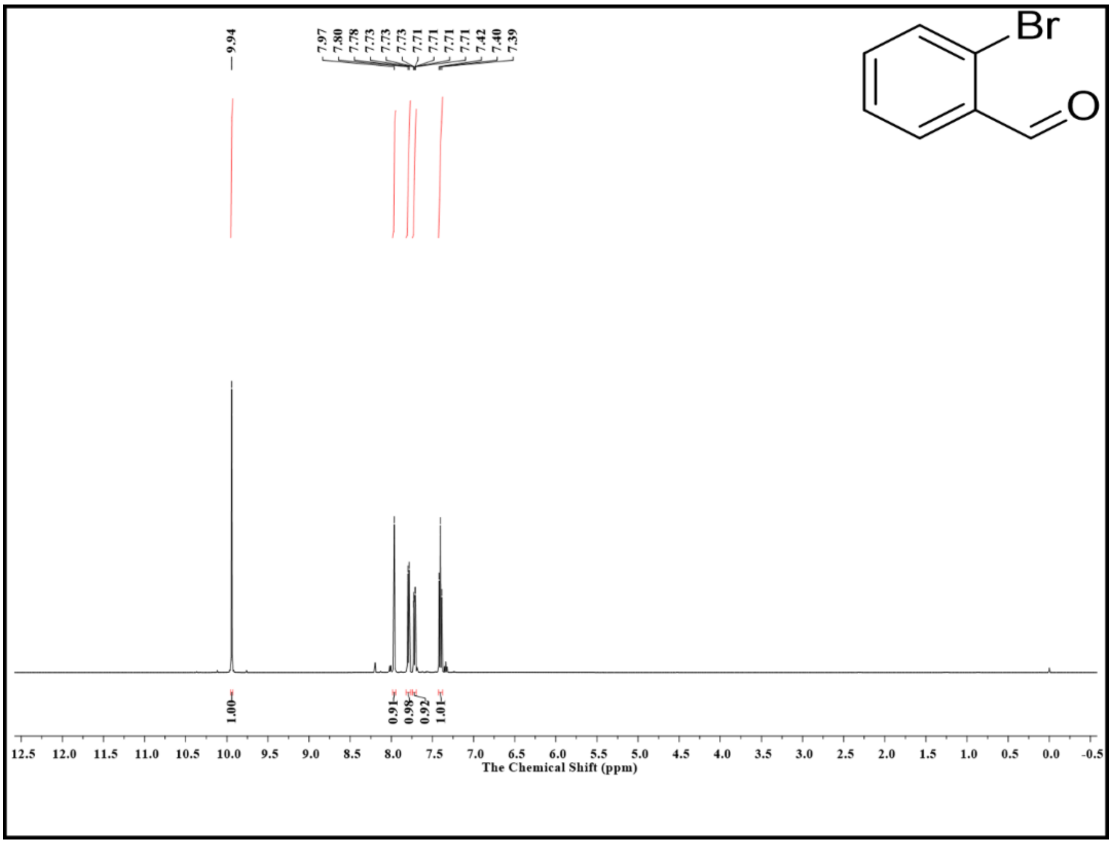

3-Bromobenzaldehyde ([13] NMR spectrum)

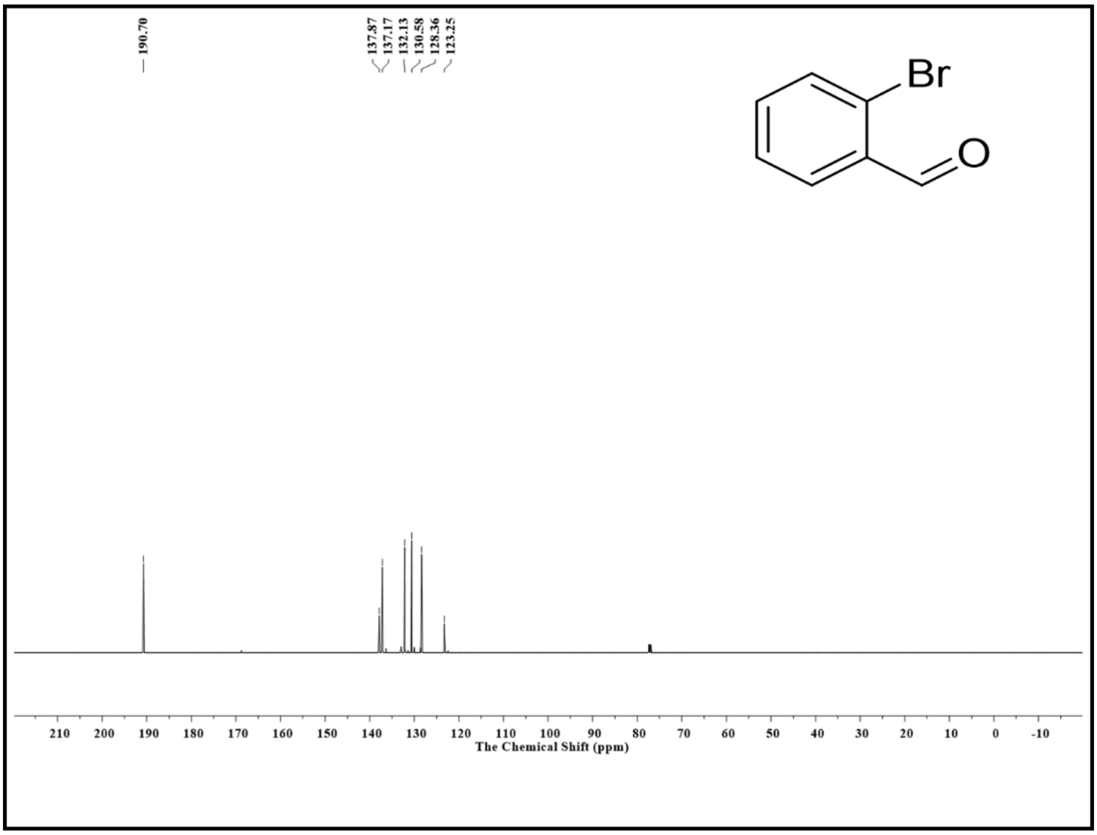

9-Anthraldehyde (^{1}H NMR spectrum)

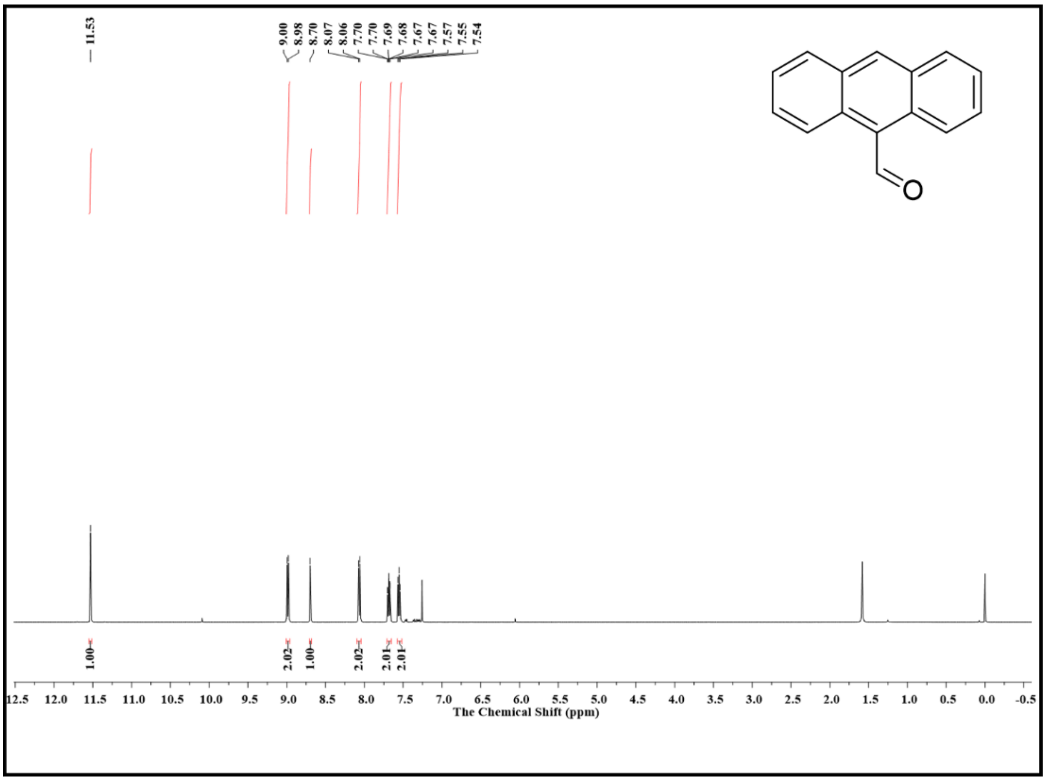

9-Anthraldehyde (^{13}C NMR spectrum)

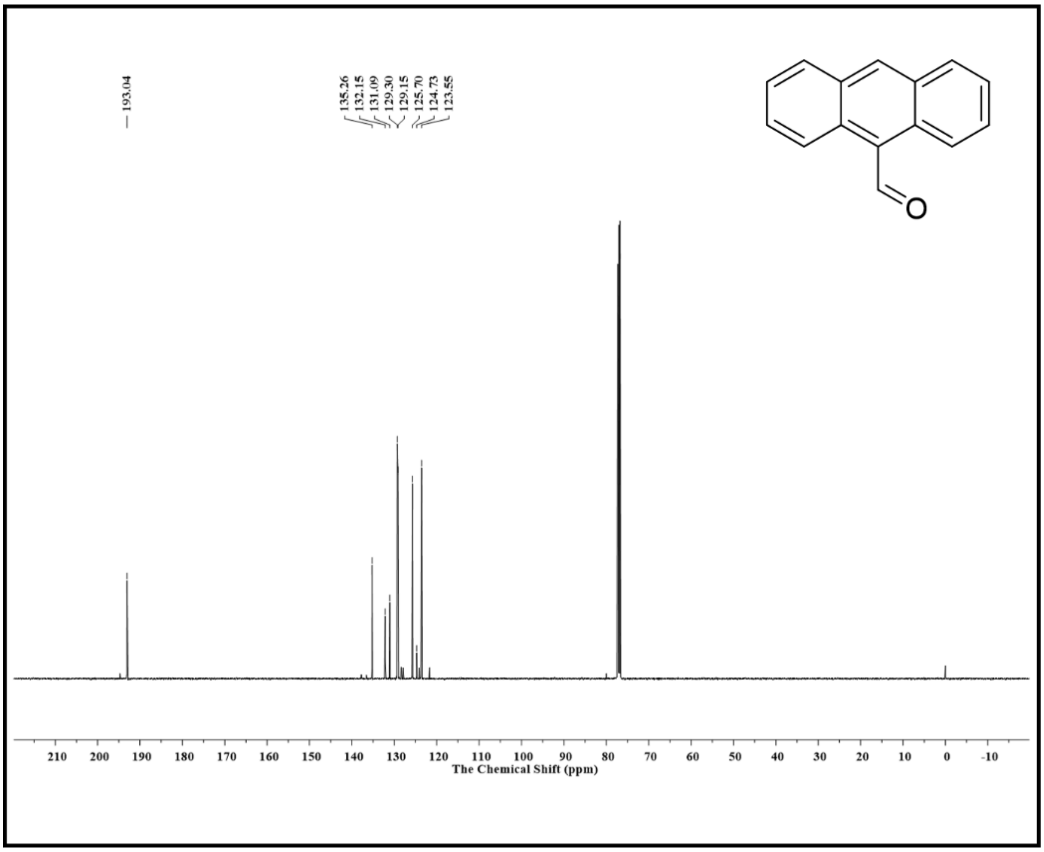

Figure S6. ^{1}H and ^{13}C NMR spectra of products.

^{1}H and [13] C NMR details of the products

Benzaldehyde: ^{1}H NMR (500 MHz, $CDCl_3$) δ 10.01 (s, 1H), 7.90–7.86 (m, 2H), 7.66–7.60 (m, 1H), 7.52 (t, *J* = 7.7 Hz, 2H). ^{13}C NMR (126 MHz, $CDCl_3$) δ (ppm) 192.42, 136.38, 134.47, 129.73, 128.99.
Cyclopentanone: ^{1}H NMR (500 MHz, $CDCl_3$) δ 2.22–2.11 (m, 1H), 2.01–1.94 (m, 1H). ^{13}C NMR (126 MHz, $CDCl_3$) δ (ppm) 220.21, 38.23, 23.14.
2-Methoxy benzaldehyde: ^{1}H NMR (500 MHz, $CDCl_3$) δ 9.95 (s, 1H), 7.45–7.40 (m, 2H), 7.37 (d, *J* = 1.8 Hz, 1H), 7.18–7.13 (m, 1H), 3.83 (s, 3H). ^{13}C NMR (126 MHz, $CDCl_3$) δ 192.16, 160.14, 137.80, 130.04, 123.44, 121.41, 112.15, 55.39.
3-Bromo benzaldehyde: ^{1}H NMR (500 MHz, $CDCl_3$) δ 9.94 (s, 1H), 7.97 (s, 1H), 7.79 (d, *J* = 7.6 Hz, 1H), 7.74–7.70 (m, 1H), 7.40 (t, *J* = 7.8 Hz, 1H). ^{13}C NMR (126 MHz, $CDCl_3$) δ 190.70, 137.87, 137.17, 132.13, 130.58, 128.36, 123.25.
9-Anthraldehyde: ^{1}H NMR (500 MHz, $CDCl_3$) δ 11.53 (s, 1H), 8.99 (d, *J* = 9.0 Hz, 2H), 8.70 (s, 1H), 8.07 (d, *J* = 8.4 Hz, 2H), 7.71–7.65 (m, 2H), 7.58–7.52 (m, 2H). ^{13}C NMR (126 MHz, $CDCl_3$) δ 193.04, 135.26, 132.15, 131.09, 129.22, 125.70, 124.73, 123.55.

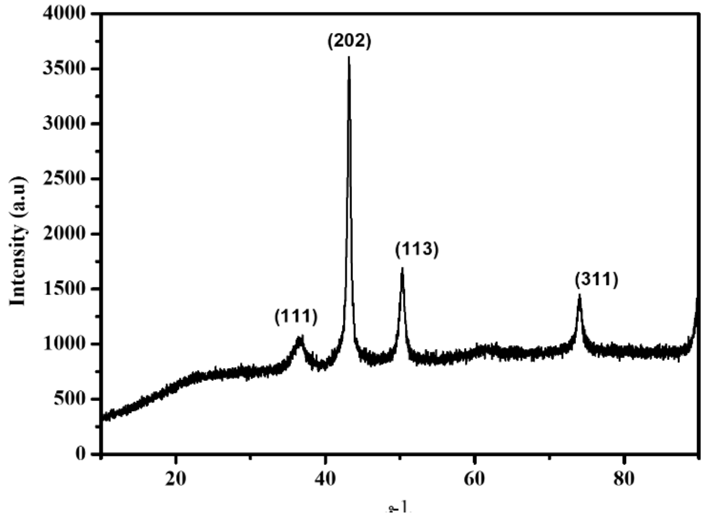

Figure S7. WAXRD patterns of CuNPs/p(DMAEMA-*co*-TBA)/TSBA hybrid catalyst on the oxidation of alcohol after fifth wash.

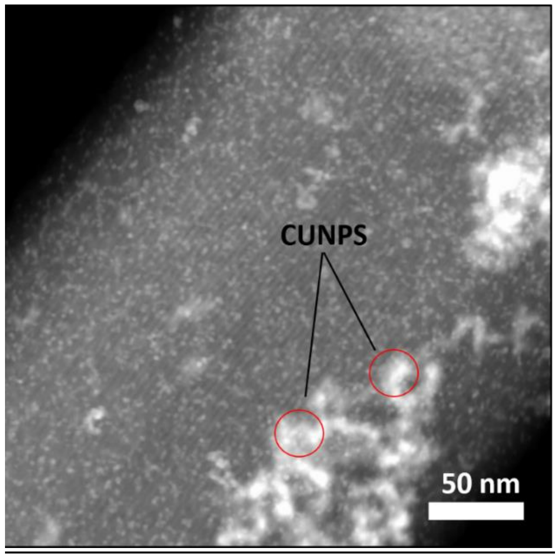

Figure S8. HRTEM image of CuNPs/p(DMAEMA-*co*-TBA)/TSBA hybrid catalyst on the oxidation of alcohol after fifth wash.

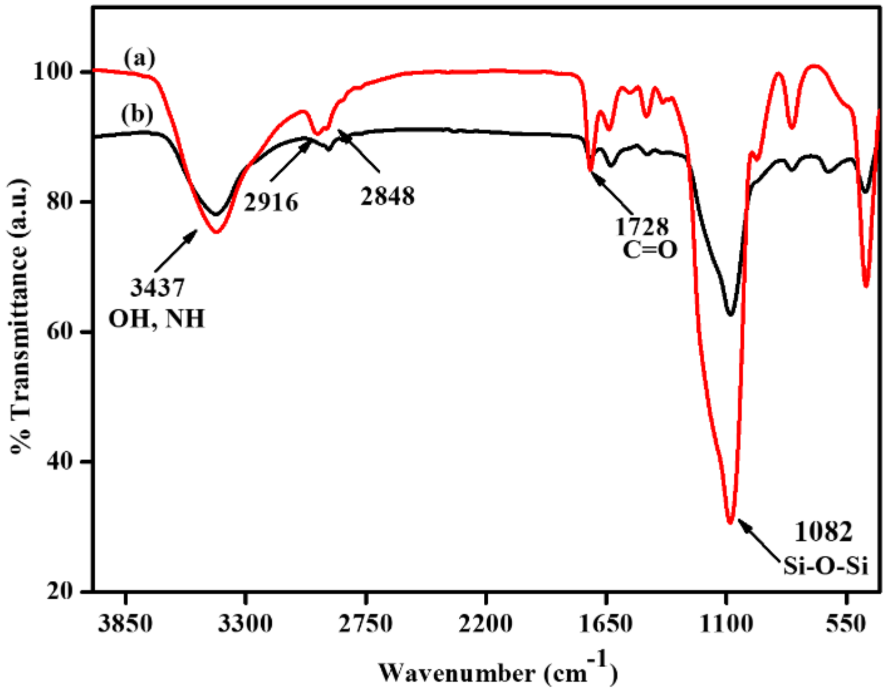

Figure S9. FT-IR spectra of CuNPs/p(DMAEMA-*co*-TBA)/TSBA hybrid catalyst on the oxidation of alcohols at (**a**) fresh and (**b**) after fifth wash.

Table S1. Brunauer–Emmett–Teller (BET) surface area, pore volume, and pore sizes distribution values of SBA 15, TSBA, and p(DMAEMA-*co*-TBA)/TSBA.

Sample	Surface area (m^2g^{-1})	Pore volume (cm^3g^{-1})	Pore size (nm)
SBA-15	708	0.89	7.7
TSBA	600	0.65	6.8
P(DMAEMA-*co*-TBA)	353	0.56	6.1

Table S2. Effect of solvent in oxidation of benzyl alcohol[a].

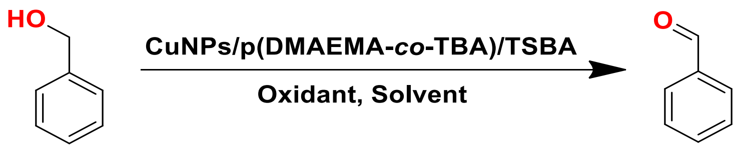

Entry	Solvent	Time (min)[b]	Conversion (%)[c]
1	DMSO	300	90
1	Acetonitrile	300	53
2	Toluene	180	90
3	n-Hexane	90	76
4	Water	45	99

[a] Reaction conditions: benzyl alcohol (1 mmol), 0.005 g catalyst, H_2O_2 (1 mmol), and 5 mL solvent at room temperature; [b] Time of maximum conversion; [c] Conversion was calculated using GC.

Table S3. Effect of oxidants in oxidation of benzyl alcohol[a].

Entry	Oxidants (mmol)	Time (min)	Conversion (%)[b]
1	*t*-BuOOH (0.5)	300	52
2	*t*-BuOOH (1)	300	60
3	H_5IO_6 (0.5)	180	81
4	H_5IO_6 (1)	120	89
5	H_2O_2 (0.5)	60	90
6	H_2O_2 (1)	45	99
7	-	300	41

[a] Reaction conditions: benzyl alcohol (1 mmol), 5 mg catalyst, and 5 mL solvent at room temperature; [b] Maximum conversion.

Table S4. Effect of catalyst amount in oxidation of benzyl alcohol[a].

Entry	Catalyst amount (g)	Conversion (%)
1	-	18
2	0.003	70
3	0.004	89
5	0.005	99
6	0.006	99

[a] Reaction conditions: benzyl alcohol (1 mmol), H_2O_2 (1 mmol), 5 mL water, 45 min at room temperature.

Table S5. Effect of different catalysts in oxidation of benzyl alcohol[a].

Entry	Catalyst	Time (min)	Conversion (%)
1	No catalyst	1440	18
2	SBA-15	300	21
3	CuNPs/SBA-15	300	80
4	p(DMAEMA)/TSBA	1440	15
5	CuNPs/p(DMAEMA)/TSBA (50)	300	86
6	CuNPs/p(DMAEMA)/TSBA (25)	300	91
7	CuNPs/p(DMAEMA)	300	60
8	p(DMAEMA-*co*-TBA)/TSBA	1440	35
9	CuNPs/p(DMAEMA-*co*-HEA)/TSBA (50)	300	91
10	CuNPs/p(DMAEMA-*co*-TBA)/TSBA (25)	45	99
11	CuNPs/p(DMAEMA-*co*-HEA)	300	65
12[b]	No catalyst	600	8

[a] Reaction conditions: benzyl alcohol (1 mmol), H_2O_2 (1 mmol), 5 mg catalyst, 5 mL water at room temperature; [b] No oxidant used.

Table S6. Total expense for the synthesis of CuNPs/p(DMAEMA-*co*-TBA)/TSBA hybrid catalyst.

Materials	Used amount (g/mL)	Unit price ($)	Total price ($)
Pluronic (poly(ethylene glycol)-block-poly(propylene glycol)-block-poly(ethylene glycol)) (P123)	16g	$0.14 (g)	$2.24
Tetraethyl orthosilicate (TEOS)	36.8 g	$0.09 (mL)	$3.31
Toluene	60 mL	$0.095 (mL)	$5.7
Hydrochloric acid (HCl)	80 mL	$0.21 (mL)	$16.8
3-trimethoxysilylpropyl methacrylate (TMM)	3 mL	$3.36 (mL)	$10.08
dimethyl aminoethyl methacrylate (DMAEMA)	0.6 g	$1.11 (mL)	$0.66
tert-butyl acrylamide (TBA),	0.4 g	$0.87 (mL)	$0.34
Copper (II) Bromide (Cu(II)Br)	0.01 g	$1.11 (g)	$0.88
potassium persulfate ($K_2S_2O_8$)	0.027 g	$0.59 (g)	$0.02
sodium borohydride ($NaBH_4$)	0.037 g	$4.33 (g)	$0.16
Ethanol	20 mL	$0.19 (mL)	$3.8

Total expense for the synthesis of 1 g of CuNPs/p(DMAEMA-co-TBA)/TSBA catalyst is $0.54 (This work).

Table S7. Total expense for the synthesis of Copper nanoparticles based on the reference [2].

Materials	Used amount (g/mL)	Unit price ($)	Total price ($)
Copper Sulphate pentahydrate ($CuSO_4.5H_2O$)	2.5 g	$10	$25
Sodium borohydride ($NaBH_4$)	3.78 g	$4.576	$17.30
Sodiumhydroxide (NaOH)	40 g	$2.368	$94.72
Ascorbic acid	3.52	$1.413	$4.97

Total expense for the synthesis of 1g of Copper nanoparticles is $56.8 [2].

Table S8. Total expense for the Synthesis of Iron nanoparticles based on the reference [3].

Materials	Used amount (g/mL)	Unit price ($)	Total price ($)
Iron(II)Chloride ($FeCl_3$)	0.0973 g	$161.70 (g)	$15.73
Iron(II)sulphate ($FeSO_4$)	1.39 g	$7.656 (g)	$10.64
Trisodium citrate	4.4 g	$0.2821 (g)	$1.211

Total expense for the synthesis of 1 g of Iron nanoparticles is $27.56. In this reference they did not mention anything about how much gram or yield of FeNPs they synthesized [3].

Table S9. Total expense for the Synthesis of Iron nanoparticles based on the reference [4].

Materials	Used amount (g/mL)	Unit price ($)	Total price ($)
Ferrous Sulphate heptatahydrate ($CuSO_4.5H_2O$)	1.39 g	$7.8 (g)	$11
Sodium borohydride ($NaBH_4$)	0.9462 g	$4.576 (g)	$4.33
PVP	11 g	$1.023 (g)	$11.83
Ethanol	20 mL	$0.19 (mL)	$3.5

Total expense for the synthesis of 1g Iron nanoparticles is $38.32 [4].

Our ultimate aim is to synthesize a switchable smart hybrid catalyst for various applications. In this work, we used the oxidation reaction as a model reaction. The main advantage of this work is that we can control the reaction rate with respect to the external stimuli. Usually, for the synthesis of these kinds of catalysts, silver or palladium nanoparticles are used. We have also checked the total expenses for the synthesis CuNPs, and FeNPs in the literature, which also showed that our catalyst is much cheaper than CuNPs and FeNPs [2–4]. It is noteworthy that in the present work, we used copper as cost-effective nanoparticles instead of those expensive metal nanoparticles, which we believe to be surely one of the significant advantages of the present work.

References

1. Adrover, M.E.; Pedernera, M.; Bonne, M.; Lebeau, B.; Bucala, B.; Gallo, L. Synthesis and Chracterization of Mesoporous SBA-15 and SBA-16 as Carriers to Improve Albendazole Dissolution Rate. *Saudi Pharm J.* **2020**, *28*, 15–24.
2. Khalid, H.; Shamaila, S.; Zafar, N. Synthesis of Copper Nanoparticles by Chemical Reduction Method. *Sci. Int. (Lahore)* **2015**, 27, 3085–3088.
3. Banerjee, A.; Yao, Y.; Durr, A.R.R.; Barrett, W.G.; Hu, Y.; Scott, R.W.J. Synthesis, Characterization, and Evaluation of Iron Nanoparticles as Hydrogenation Catalysts in Alcohols and Tetraalkylphosphonium Ionic Liquids: Do Solvents Matter? *Catal. Sci. Technol.* **2018**, *8*, 5207–5217.
4. Viju, K.V.G.; Prem, A.A. Green Synthesis and Characteristics of Iron Oxide Nanoparticles using Phyllanthus Niruri extract. *Orient. J. Chem.* **2018**, *34*, 2583–2589.
